# The Use of Botulinum Toxin Injections in Peripheral Neuropathic Pain: A Systematic Review of Efficacy and Safety Outcomes

**DOI:** 10.1155/prm/7701940

**Published:** 2026-01-16

**Authors:** Domenico Antonio Restivo, Andrea Calderone, Angelo Quartarone, Rocco Salvatore Calabrò, Antongiulio Bruschetta

**Affiliations:** ^1^ Department of Clinical and Experimental Medicine, Physical Medicine and Rehabilitation Unit, University of Messina, Messina, Italy, unime.it; ^2^ IRCCS Centro Neurolesi Bonino-Pulejo, Messina, Italy; ^3^ Orthopaedic Institute of Southern Italy “Franco Scalabrino”, Messina, Italy

**Keywords:** botulinum toxin, efficacy, injection techniques, neurotransmitter release, pain management, peripheral neuropathic pain, safety

## Abstract

Peripheral neuropathic pain (PNP), a chronic condition resulting from nerve damage and characterized by altered sensory signaling and central sensitization, poses significant therapeutic challenges. Botulinum toxin (BTX), known for neuromuscular blockades, also exhibits analgesic properties, prompting its investigation for PNP management. However, existing evidence regarding its efficacy and safety is fragmented. This systematic review aimed to synthesize current data on BTX injections for PNP. Guided by PRISMA and the neuromatrix theory, major databases (PubMed, Web of Science, Cochrane, Embase, Scopus, EBSCOhost) were searched up to March 2025. Included studies evaluated BTX (primarily type A) in adults with PNP using validated pain outcomes and reported safety, covering designs from randomized controlled trials (RCTs) to observational research. Independent reviewers performed data extraction and quality assessment using risk of bias tools. This review has been registered on Prospero with the following number: CRD420251022222. The patient groups studied (1343 participants: 547 males and 632 females) showed substantial variability in age, diagnosis, and treatment setting. Results indicated BTX use across diverse PNP etiologies (e.g., trigeminal neuralgia, painful diabetic neuropathy (DN), postherpetic neuralgia, and phantom limb pain) and across multiple countries. RCTs often reported statistically significant reductions in pain intensity and improvements in related outcomes compared with placebo, although effect sizes were heterogeneous and sample sizes were generally modest. Nonrandomized studies suggested similar trends but frequently presented moderate to serious risk of bias. Adverse events were usually mild and transient, most often localized injection‐site reactions or temporary facial asymmetry in cranial applications, while serious complications were rare but could not be excluded with confidence because of incomplete safety reporting. Overall, BTX may offer clinically meaningful benefit for selected PNP subtypes, particularly trigeminal neuralgia and painful DN, yet the certainty of evidence remains low to moderate due to study limitations and methodological diversity. Routine use in PNP therefore requires cautious, individualized consideration rather than broad generalization.

**Highlights**

- Botulinum toxin A (BTX‐A) shows moderate potential benefit for several peripheral neuropathic pain disorders, particularly focal and refractory conditions.
- Clinically relevant pain reduction is most consistently reported in trigeminal neuralgia and diabetic peripheral neuropathy, although effect sizes and responder rates vary across trials.
- The safety profile of BTX‐A is generally favorable, with most adverse events being mild and transient, while rare but relevant adverse events require careful monitoring and patient counseling.

## 1. Introduction

According to the International Association for the Study of Pain (IASP), neuropathic pain (NP) is defined as pain initiated or caused by a primary lesion or dysfunction in the nervous system [1]. Its management is challenging because responses to many drugs are often unpredictable [2]. Peripheral neuropathic pain (PNP) arises from a lesion or disease affecting the peripheral somatosensory nervous system [3]. In older adults, its prevalence varies widely, from 0.2% to 10% in people over 50 years [4]. PNP is sustained by central sensitization mechanisms [5, 6] and may result from several conditions affecting the peripheral nervous system, including diabetic neuropathy (DN) [7], postherpetic neuralgia (PHN) [8], nerve compression [9], post‐traumatic or postoperative neuropathy [10], and chemotherapy‐induced neuropathy [11]. Clinically, NP presents as a combination of negative and positive sensory symptoms. Negative symptoms such as hypoesthesia and anesthesia reflect loss of sensory function, whereas positive symptoms, including allodynia and hyperalgesia, denote abnormal pain perception in response to normally nonpainful or mildly painful stimuli [12, 13]. These symptom types often co‐occur in the same innervation territory, complicating diagnosis and treatment [14]. Botulinum toxin type A (BTX‐A) is a potent neurotoxin produced by *Clostridium botulinum* and has long been used as an effective treatment for focal muscular hyperactivity, particularly dystonia and spasticity, by blocking presynaptic acetylcholine (ACh) release at the neuromuscular junction (NMJ) [15, 16]. Early clinical observations in patients with dystonia suggested that BTX‐A might provide pain relief that preceded and exceeded the expected benefit from muscle relaxation [17, 18], raising the hypothesis that BTX‐A exerts direct analgesic actions independent of neuromuscular blockade. Experimental work supports this hypothesis: In vitro studies show that BTX‐A inhibits neurogenic inflammation by modulating C‐fiber nociceptor sensitization [19] and reduces the release of pro‐algogenic neurotransmitters such as calcitonin gene‐related peptide (CGRP), substance P, and glutamate, while also inhibiting vanilloid receptor activity [20–22]. Peripheral BTX‐A injections reduce pain behaviors in animal models of inflammatory and traumatic NP [23–25]. Taken together, these findings indicate that BTX‐A can modulate NP, particularly pain related to peripheral nerve lesions associated with allodynia and hyperalgesia. The first double‐blind, placebo‐controlled clinical trial by Ranoux et al. [10] reported that intradermal BTX‐A injections into painful areas with spontaneous and allodynic pain improved symptoms in patients with focal NP, including post‐traumatic/postoperative pain and PHN [10]. The treatment was well tolerated, and no relevant changes in muscle tone were observed [10]. Importantly, the analgesic effects of BTX‐A appear restricted to positive sensory symptoms (spontaneous pain, allodynia, hyperalgesia), with no effect on hypoesthesia or anesthesia (negative sensory symptoms) [10, 26, 27]. Based on these results, BTX‐A has been included in the European Federation of Neurological Societies (EFNS) recommendations for NP management [28]. Other early studies evaluated BTX‐A in PHN and additional NP conditions, including DN, diabetic meralgia paresthetica, NP related to phantom limb pain (PLP) [29, 30], and muscle cramps associated with DN [31]. In the latter condition, Restivo et al. [31] suggested that pain reflects a combination of neuropathic and ischemic/non‐neuropathic mechanisms, with overlapping muscular and nonmuscular components, implying a dual mechanism of action for BTX‐A. BTX‐A has also been explored in painful conditions that predominantly involve small fibers, such as burning mouth syndrome (BMS), where preliminary data suggest meaningful pain relief [32, 33].

### 1.1. Gap in Literature, Rationale, and Objective

Although BTX‐A has received growing interest as a possible treatment for PNP, the available clinical literature remains fragmented and often difficult to interpret. Individual trials have tested heterogeneous protocols, enrolled small and clinically diverse samples, and reported variable outcomes. A coherent synthesis that jointly evaluates efficacy, safety, and the practical aspects of patient selection and injection strategies is still missing. Existing reviews have already highlighted a potential role for BTX‐A in specific conditions, such as painful DN or focal neuropathic syndromes, but most focus on single etiologies or include only randomized trials, limiting their ability to capture the broader spectrum of clinical use. Recent systematic reviews and meta‐analyses, for example in chronic PNP and in painful DN, confirmed that BTX‐A can reduce pain intensity without a clear increase in adverse events, yet they also emphasized that the evidence base is modest and that BTX‐A remains a third‐line option in major NP treatment algorithms [34, 35]. Recent work focused on orofacial NP further underlined that BTX‐A represents a promising but still emerging tool that should be positioned cautiously within multidisciplinary care pathways rather than replacing established first‐line pharmacological options [36, 37]. More detailed, methodical evaluation is therefore needed to clarify which PNP phenotypes benefit most, which dosing and injection techniques are most consistently associated with response, and how durable and safe these effects are over time. The rationale for this systematic review is to address these gaps and to provide clinicians and patients with an updated, evidence‐based overview of BTX‐A efficacy and safety across the main PNP subtypes. For these reasons, this systematic review aims to synthesize current evidence regarding BTX‐A injections in PNP, with explicit attention to NP phenotype, injection protocol, and risk of bias. The review addresses the following questions: (i) What is the overall efficacy of BTX‐A injections in reducing pain intensity and improving functional outcomes in adults with PNP?; (ii) How does efficacy vary across different peripheral neuropathic etiologies such as painful DN, PHN, trigeminal neuralgia, PLP, complex regional pain syndrome (CRPS), and other focal neuropathies?; and (iii) What adverse events are reported with BTX‐A injections in this setting, and how robust is the safety evidence?

Recent systematic reviews and meta‐analyses confirm that BTX‐A injections can significantly reduce pain in patients with PNP, especially painful diabetic polyneuropathy, without a clear increase in adverse events [34, 35]. However, these analyses focus mainly on selected etiologies and strictly randomized designs. The present review is designed to complement and extend that work by integrating randomized and nonrandomized evidence across a wider range of PNP phenotypes and by linking efficacy signals with safety data, injection techniques, and risk‐of‐bias profiles. Mechanistic studies suggest that BTX‐A may modulate pain not only through its classical action at the NMJ but also through inhibition of pain‐facilitating neuropeptides such as substance P and CGRP, with consequent reduction in neurogenic inflammation and peripheral sensitization [20–24, 38]. Experimental and translational data also indicate a possible influence on central sensitization, which may help explain durable benefit in some neuropathic syndromes [12, 24, 38]. These converging pieces of evidence support a careful, mechanism‐informed exploration of BTX‐A as a targeted option for selected PNP conditions rather than a universal analgesic approach. Table 1 provides an overview of the different modalities of BTX‐A injection in NP treatment, offering a deeper understanding of its application and therapeutic potential [38–54].

**Table 1:** Modalities of BTX injection in neuropathic pain treatment (cited at the end of the Introduction).

Injection method	Description	Targeted conditions	Mechanism of action	Advantages	Limitations	Clinical considerations
Intradermal [38]	Superficial injection into the dermis, typically at multiple sites in affected areas.	Small fiber neuropathy, burning mouth syndrome, localized allodynia	Blocks local sensory nerve endings, reducing the release of neuropeptides like substance P and CGRP	Precise targeting, minimal systemic diffusion, low risk of side effects	Shorter duration of effect, requires frequent re‐injections, may not reach deeper nerve structures	Best for localized pain; often used in facial or hand neuropathy.
Subcutaneous [39–43]	Injection into the subcutaneous layer just beneath the skin	Postherpetic neuralgia, diabetic neuropathy, trigeminal neuralgia	Reduces neurogenic inflammation and alters peripheral sensitization	Simple administration, well tolerated, useful for focal pain	Limited penetration to deeper nerves, less effective for widespread neuropathic pain	Suitable for patients who prefer less invasive procedures; may require multiple sites
Perineural [44]	Injection near affected peripheral nerves, often under ultrasound guidance	Trigeminal neuralgia, post‐traumatic neuropathy, chemotherapy‐induced neuropathy	Modulates abnormal nerve firing, reduces ectopic discharge from damaged nerves	Direct effect on the nerve, prolonged pain relief compared to superficial injections	Requires imaging guidance for accuracy, with a potential risk of nerve damage if not precisely administered	Ideal for mononeuropathies; may need combination therapy with systemic analgesics
Intramuscular [45]	Injection into affected muscle groups, often in areas of spasm or tension	Myofascial pain, spasticity‐related neuropathic pain, temporomandibular disorders	Reduces excessive muscle contraction, alleviates secondary nerve compression, inhibits pain‐related neurotransmitters	Effective for pain linked to muscle hyperactivity, longer duration of action than superficial injections	Less effective for purely neuropathic conditions, potential for muscle weakness in unintended areas	Common in poststroke spasticity and dystonic conditions; requires dose titration
Intra‐articular [46–49]	Injection directly into synovial joints to modulate joint‐related pain	Neuropathic pain secondary to osteoarthritis, inflammatory arthritis, post‐traumatic joint pain	Reduces joint inflammation, blocks synovial nerve endings from transmitting pain	Targets joint‐related pain, minimally invasive, potential to delay need for systemic analgesics	Limited data on long‐term efficacy in neuropathic pain, may not address central pain sensitization	Often combined with corticosteroids or HA injections in arthritic conditions
Epidural [50]	Injection into the epidural space surrounding the spinal cord	Neuropathic pain from radiculopathy, spinal cord injury, failed back surgery syndrome	Inhibits abnormal pain signaling in spinal nerve roots, reduces central sensitization	Provides broader pain relief in affected dermatomes and longer‐lasting effects than peripheral injections	Requires expertise and potential for systemic diffusion leading to side effects like weakness or autonomic dysfunction	May be considered in refractory pain cases, particularly when other treatments fail
Intrathecal [51]	Injection directly into CSF for central action	Central neuropathic pain, refractory pain syndromes, complex regional pain syndrome	Modifies pain pathways at the spinal cord level, reduces pain signal transmission	Direct CNS action bypasses peripheral barriers and is effective for intractable pain	High risk of adverse effects, requires hospitalization and specialized care, potential for severe complications like meningitis	Reserved for severe, drug‐resistant cases; requires careful patient selection
Ultrasound‐guided [52–54]	Injection performed under real‐time ultrasound imaging to improve precision	Neuropathic pain affecting deep nerves, difficult‐to‐access anatomical regions	Enhances accuracy in localizing target nerves, minimizing the risk of off‐target injections	Improved safety and effectiveness, reduces complications, ensures precise targeting	Requires specialized training and equipment, higher cost compared to blind injections	Useful for deep nerve blocks or difficult anatomical regions (e.g., pelvic pain)

Abbreviations: CGRP, calcitonin gene‐related peptide; CSF, cerebrospinal fluid; HA, hyaluronic acid.

## 2. Materials and Methods

### 2.1. Inclusion Criteria

In this systematic review we considered studies that aimed to evaluate the effectiveness and safety of BTX‐A treatment for PNP management. Included studies investigated BTX‐A as a therapeutic intervention in adult individuals aged 18 years or older who had previously received a diagnosis of PNP. This encompassed, but was not limited to, DN, TN, PHN, and CRPS. In addition, studies were included if they assessed pain‐related outcomes (i.e., pain intensity or pain relief) using established and validated pain assessment tools and if they reported on the safety profile of BTX‐A injections (i.e., adverse events). The settings of the included studies ranged widely across healthcare settings. Including but not limited to hospital‐based clinics, specialty pain management facilities, outpatient care settings, and private practices. Owing to this, geographical location did not serve as an exclusion criterion, and thus a thorough synthesis of global evidence was enabled. Such study designs included randomized controlled trials (RCTs), double‐blind trials, open‐label trials, pilot trials, non‐RCT experimental studies, cohort studies, cross‐sectional studies, retrospective studies, observational studies, feasibility studies, and long‐term studies to provide a complete analysis of the evidence available. Additionally, we included studies that had clear and detailed descriptions of the BTX‐A treatment, such as dosage, injection sites, and methods of administration. Lastly, only full‐text articles published in English were included, acknowledging the possibility of language bias and the potential omission of relevant studies published in other languages.

### 2.2. Exclusion Criteria

To maintain methodological rigor and relevance, some exclusion criteria were employed. Studies that did not specifically investigate the use of BTX‐A injections for the management of PNP were excluded. Thus, studies with interventions other than BTX‐A or involving the management of pain conditions of nonperipheral origin (i.e., NP of central origin) or that did not use a study population entirely made up of subjects with PNP were excluded. Studies that did not clearly state the PNP diagnosis as a term of inclusion were also excluded. Additionally, based on the purpose of the review, study protocols, case studies, development studies, and reviews (including systematic, narrative, and integrative reviews) were also not included. Studies based on animal models were also excluded. Finally, studies that did not provide adequate detail on the BTX‐A injection protocols, including dosage, injection sites, and administration technique, were not included in the review to highlight clinically relevant and adequately described practices.

### 2.3. PICO Evaluation

We applied the PICO model (Population, Intervention, Comparison, Outcome) to create our search terms [55, 56]. The population includes individuals suffering from PNP conditions such as TN, DN, PHN, PLP, CRPS, and other related disorders. The intervention under evaluation is the administration of BTX‐A through various routes, including intradermal, subcutaneous, intramuscular, and bladder inoculation. The comparative analyses within the included studies involve placebos, alternative treatments like lidocaine/depomedrol injections, conventional oral therapies, and variations in BTX‐A dosages or types. The primary outcome measures are focused on the reduction of pain intensity, assessed through validated pain scales, while secondary outcomes evaluate improvements in quality of life, sleep quality, allodynia, cramps, synkinesis, and the overall safety profile of BTX‐A treatments.

### 2.4. Search Strategy

We followed a predefined, transparent approach to identify eligible studies. Searches were run from March 3, 2025, through March 31, 2025, with no lower date boundary so as not to exclude earlier, pertinent work. We set March 31, 2025, as the endpoint ensured coverage of the most recent literature available at the time. We queried PubMed, Web of Science, the Cochrane Library, Embase, EBSCOhost, and Scopus to maximize disciplinary breadth across biomedicine, clinical trials, and allied health. The strategy combined controlled vocabulary and free‐text terms for BTX and NP using Boolean operators: (“botulinum toxins”[MeSH Terms] OR (“botulinum”[All Fields] AND “toxins”[All Fields]) OR “botulinum toxins”[All Fields] OR (“botulinum”[All Fields] AND “toxin”[All Fields]) OR “botulinum toxin”[All Fields]) AND (“neuralgia”[MeSH Terms] OR “neuralgia”[All Fields] OR (“neuropathic”[All Fields] AND “pain”[All Fields]) OR “neuropathic pain”[All Fields]). Databases were selected to balance sensitivity and specificity across clinical and mechanistic studies relevant to this review.

#### 2.4.1. Data Extraction

**Figure 1:**
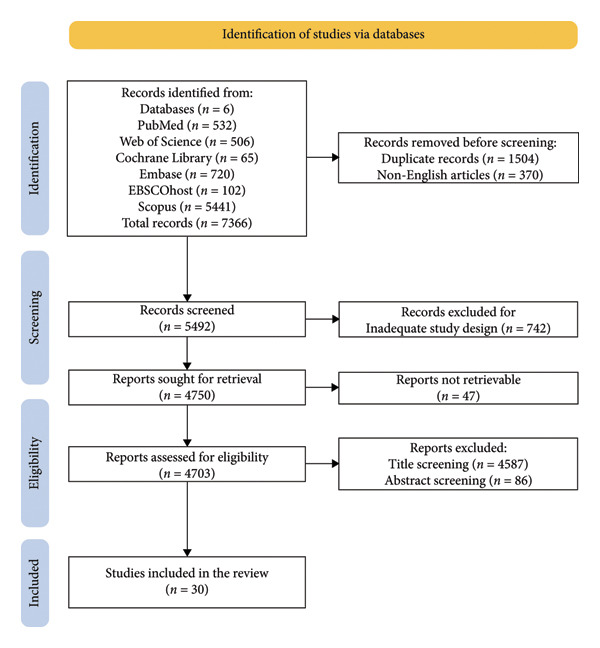
PRISMA 2020 flow diagram of evaluated studies.

Two reviewers (Domenico Antonio Restivo and Andrea Calderone) worked in parallel to identify and evaluate eligible studies, a workflow designed to enhance transparency and reproducibility. The information‐retrieval strategy was piloted and then tuned across iterations by varying keyword groupings, Boolean logic, and controlled vocabulary (e.g., MeSH) to balance sensitivity with precision. Study selection was documented in a PRISMA diagram summarizing identification, screening, eligibility, and inclusion (Figure 1) [57]. Titles/abstracts and subsequently full texts were screened, and data were extracted independently to limit bias from missing results, publication, time lag, and language effects. Extracted fields included study design, sample size and demographics, PNP phenotype, botulinum neurotoxin regimen (formulation, dose, and injection sites/technique), duration of follow‐up, prespecified pain outcomes, responder definitions, and adverse events. Disagreements at any stage were resolved through discussion; when consensus could not be reached, a third reviewer (Rocco Salvatore Calabrò) adjudicated. Inter‐rater reliability for inclusion decisions was quantified using Cohen’s kappa, with values > 0.61 interpreted as indicating substantial agreement in line with established thresholds [58, 59]. Data were curated in Microsoft Excel using piloted extraction templates with validation checks; tagging, filters, and sort functions supported consistent coding, audit trails, and cross‐verification. Finally, studies were organized and synthesized according to the a priori inclusion/exclusion criteria and thematic relevance. The protocol was prospectively registered with PROSPERO (CRD420251022222), providing a public record of planned methods and reinforcing methodological transparency [60, 61].

#### 2.4.2. Data Synthesis

We synthesized the evidence using a structured narrative approach complemented by targeted quantitative summaries. This strategy was chosen to accommodate the substantial clinical and methodological heterogeneity among the included studies and the diversity of NP phenotypes [62]. Studies were first grouped by design (randomized versus nonrandomized), then by NP subtype and injection approach (formulation, dose, site, and technique). Within each group, we mapped the direction and magnitude of effects for prespecified pain and functional outcomes at comparable follow‐up times. Where at least two randomized trials reported sufficiently similar interventions and outcome measures, we described the range of effect estimates but did not force formal meta‐analysis when assumptions of homogeneity were not met. This approach aligns with current guidance for synthesis without meta‐analysis (SWiM) and follows key reporting elements proposed by the SWiM statement [63], including transparent description of grouping decisions, handling of heterogeneity, and judgment of overall effect direction. Synthesis was conducted by a multidisciplinary team with expertise in pain medicine, neurology, and evidence synthesis; calibration meetings and consensus discussions were planned a priori to standardize coding rules, minimize subjective drift in judgment, and align interpretation across reviewers [64, 65]. By integrating qualitative interpretation with structured quantitative summaries, this framework provides a coherent and cautious overview of BTX‐A efficacy and safety while explicitly acknowledging areas of uncertainty.

### 2.5. Evaluating the Quality of Evidence: A Risk of Bias Approach

To systematically determine the certainty of evidence for each reported outcome, we employed the Cochrane Risk of Bias tool, while the Risk of Bias in Non‐randomized Studies – of Interventions (ROBINS‐I) tool was applied to uncontrolled experimental studies [66, 67]. This comprehensive evaluation involved examining critical aspects such as sequence generation, allocation concealment, blinding of participants and personnel, blinding of outcome assessment, completeness of outcome data, and the possibility of selective reporting. Any identified methodological limitations that could introduce bias were carefully documented and factored into the overall quality rating. Table 2 provided an overview of the methodology employed for this systematic review.

**Table 2:** Detailed summary of the systematic review methodology (cited at the end of the Methods section).

Section methodology	Details
Inclusion criteria	Participants: Adults aged 18 years or older diagnosed with peripheral neuropathic pain. Treatment: Studies investigating botulinum toxin injections, particularly botulinum toxin type A, as a therapeutic intervention. Diagnoses: Includes, but is not limited to, diabetic neuropathy, trigeminal neuralgia, postherpetic neuralgia, posttraumatic/postoperative neuropathy, and complex regional pain syndrome. Outcome measures: Studies evaluating pain‐related outcomes using established and validated assessment tools and reporting on the safety profile, including adverse events. Clinical settings: Research conducted in various healthcare environments, including hospitals, specialized pain management centers, ambulatory care settings, and private practices. No restrictions based on geographical location. Study types: A broad range of study designs, including randomized controlled trials, double‐blind trials, open‐label studies, pilot studies, nonrandomized experimental studies, cohort studies, cross‐sectional analyses, retrospective studies, observational research, feasibility studies, and longitudinal investigations. Intervention details: Only studies providing detailed descriptions of botulinum toxin administration, including dosage, injection sites, and techniques, were considered. Language requirement: Only full‐text articles published in English were included, acknowledging the possibility of language bias and the potential omission of relevant studies published in other languages.

Exclusion criteria	Pain type: Research focusing on pain conditions not of peripheral origin, including neuropathic pain of central origin. Participant criteria: Studies that failed to clearly define participant inclusion criteria, particularly those lacking a precise diagnosis of peripheral neuropathic pain. Study design: Excluded study types included study protocols, case reports, development studies, and reviews (systematic, narrative, and integrative). Animal studies: Research involving animal models or populations not exclusively comprised of individuals with peripheral neuropathic pain. Intervention details: Studies lacking sufficient details on botulinum toxin injection protocols, including dosage, injection sites, or administration techniques.

PICO evaluation [55, 56]	Population: Individuals suffering from peripheral neuropathic pain conditions such as trigeminal neuralgia, diabetic neuropathy, postherpetic neuralgia, posttraumatic/postoperative neuropathies, phantom limb pain, complex regional pain syndrome, and other related disorders. Intervention: Administration of botulinum toxin, primarily botulinum toxin type A, through various routes, including intradermal, subcutaneous, intramuscular, and bladder instillations. Comparison: Placebos, alternative treatments like lidocaine/depomedrol injections, conventional oral therapies, and variations in botulinum toxin dosages or types. Outcome: Focus on the reduction of pain intensity, assessed through validated pain scales, while secondary outcomes evaluate improvements in quality of life, sleep quality, allodynia, cramps, synkinesis, and the overall safety profile of botulinum toxin injections.

Search period	Search time range: No specific search time range. Time search conduction: From March 3, 2025, to March 31, 2025.

Study selection	Two investigators (DAR, AC) performed dual, independent screening at the title/abstract and full‐text stages; disagreements were resolved by consensus or, when required, adjudicated by a third reviewer (RSC). Study selection is depicted in a PRISMA flow diagram [57].

Tool used	Inter‐rater agreement was quantified using the kappa statistic; values > 0.61 were interpreted as indicating substantial concordance [58, 59]. Risk of bias was appraised with RoB 2 for randomized trials and ROBINS‐I for nonrandomized evidence [66, 67]. Study descriptors, RoB judgments, and outcomes were captured in a piloted Microsoft Excel workbook with data‐validation rules to limit transcription errors.

Data extraction	‐ Two reviewers (DAR, AC) independently screened and extracted data to maximize transparency and accuracy; disagreements were resolved by discussion and, when necessary, by third‐reviewer adjudication (RSC). ‐ Study selection is documented in a PRISMA diagram summarizing identification, screening, eligibility, and inclusion steps [][57]. ‐ Standardized extraction forms were used to record study design, participants, interventions, outcomes, follow‐up, and adverse events. ‐ Excel functions (tagging, filters, and audit trails) supported consistency checks and error control.

Synthesis approach	‐ Given heterogeneity in designs and interventions, we employed a narrative synthesis, with targeted quantitative summaries where outcomes were comparable [62]. ‐ Studies were organized by injection approach, neuropathic pain phenotype, and outcome domain to highlight convergent findings and important divergences. ‐ Where at least two randomized trials reported sufficiently similar interventions and outcome measures, we provided targeted quantitative summaries by describing the range of effect estimates, without forcing formal meta‐analysis when assumptions of homogeneity were not met (SWiM analysis) [63]. ‐ A multidisciplinary team conducted calibration and consensus meetings to harmonize coding rules, minimize bias, and ensure consistent interpretation across reviewers [64, 65].

Abbreviations: PRISMA, Preferred Reporting Items for Systematic Reviews and Meta‐Analyses; RoB 2, risk of bias 2 tool for randomized controlled trials; ROBINS‐I, Risk of Bias in Nonrandomized Studies – of Interventions; SWiM, synthesis without meta‐analysis.

## 3. Results

We queried six electronic databases and identified 7,366 records. After deduplication (*n* = 1,504) and exclusion of non‐English items (*n* = 370), 5,492 unique citations proceeded to title/abstract screening. We removed 742 records for design ineligibility and sought full texts for 4,750 reports; despite outreach to corresponding authors, library requests, open‐access searches, institutional resources, and professional networks, 47 could not be obtained. The remaining 4,703 reports underwent eligibility appraisal. Of these, 4,587 were excluded at the title level and a further 86 at abstract review, yielding 30 studies for inclusion (20 randomized trials and 10 nonrandomized), as depicted in Figure 1.

### 3.1. Quality of Included Studies—Risk of Bias

To appraise methodological rigor, we applied validated, design‐appropriate risk‐of‐bias tools uniformly across all included studies [68–95].

Using prespecified decision rules, we examined domains such as randomization/allocation, deviations from intended interventions, blinding and outcome assessment, missing data, and selective reporting, and judged their likely impact on effect estimates. This structured evaluation yielded a reproducible profile of each study’s vulnerabilities and strengths, providing a transparent basis for interpreting findings and situating conclusions within the overall body of evidence.

#### 3.1.1. Cochrane Risk‐of‐Bias Tool for Randomized Trials (RoB 2)

**Figure 2:**
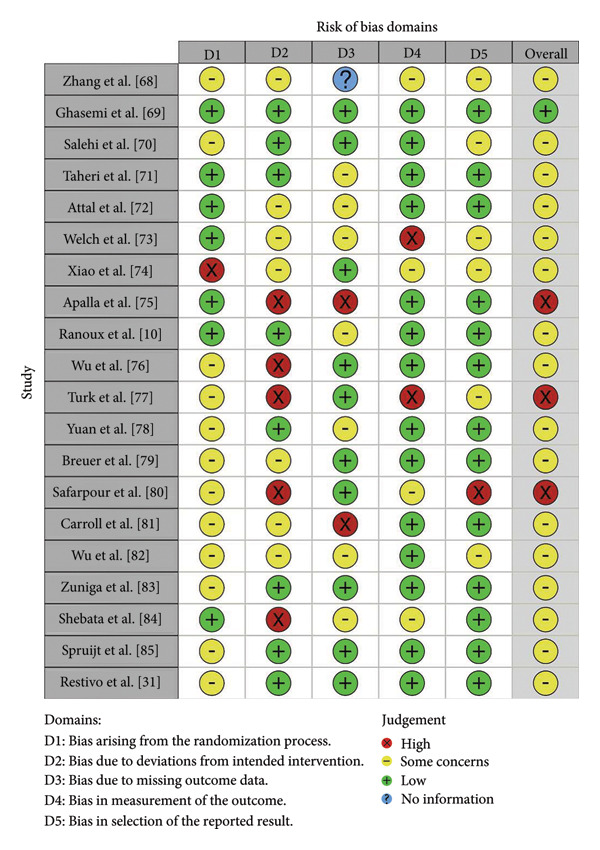
Risk of bias (RoB) of included RCT studies.

Among the 30 studies, 20 were RCTs; accordingly, we evaluated them using the revised Cochrane Risk of Bias tool (RoB 2), which considers five domains: (i) the randomization/allocation process; (ii) deviations from intended interventions; (iii) missing outcome data; (iv) outcome measurement; and (v) selection of reported results (Figure 2) [66].

The RoB 2 analysis showed marked variability in methodological rigor across the randomized trials. Only one study, by Ghasemi et al. [69], met criteria for low risk of bias in all domains. Most trials were judged as having “some concerns,” and several were rated at high risk of bias, particularly in relation to deviations from intended interventions, blinding of participants and outcome assessors, and incomplete outcome data. These issues were evident in studies by Zhang et al. [68], Salehi et al. [70], Apalla et al. [75], Wu et al. [76], Türk et al. [77], Safarpour et al. [80], and others, where limited blinding procedures, high attrition, or insufficient reporting could have affected estimated treatment effects. Nonrandom loss to follow‐up, as observed for example in the trial by Welch et al. [73], may have introduced imbalance between groups and skewed outcomes. Unclear information on outcome assessment, as in Zhang et al. [68], and recurring concerns regarding selective reporting of results further reduced confidence in some findings [68, 70, 96]. These limitations indicate that the apparent benefits of BTX‐A in several RCTs should be interpreted with caution and underscore the need for more rigorous trial design and reporting.

#### 3.1.2. The ROBINS‐I

**Figure 3:**
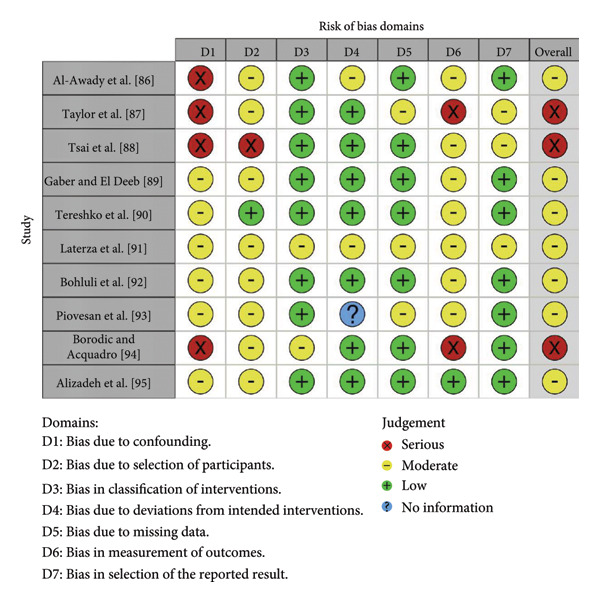
Cochrane Risk of Bias in Nonrandomized Studies – of Interventions (ROBINS‐I).

The ten nonrandomized investigations comprised one retrospective analysis [86], five single‐arm experimental reports [87, 91, 93–95], two prospective cohorts [88, 92], one comparative study [89], and one observational study [90]. We evaluated risk of bias with ROBINS‐I across seven prespecified domains: confounding, selection of participants, intervention classification, deviations from intended interventions, missing data, outcome measurement, and selective reporting (Figure 3) [67] and synthesized domain ratings to derive an overall study‐level judgment.

The ROBINS‐I evaluation identified a consistent pattern of moderate to serious risk across several domains in the nonrandomized studies. Only a minority of reports achieved an overall judgment of moderate risk, while many were rated as serious in key domains such as confounding, selection of participants, and deviations from intended interventions [86–88, 91, 93–95]. These concerns reflect limited control of baseline differences, unclear criteria for treatment allocation, and insufficient reporting of cointerventions, all of which can distort observed treatment effects. Missing data and incomplete follow‐up further complicated interpretation in some studies, including the analysis by Piovesan et al. [93], where attrition could not be adequately assessed. The cumulative impact of these methodological weaknesses means that nonrandomized evidence can only support exploratory conclusions, and any apparent improvement with BTX‐A must be considered hypothesis‐generating rather than confirmatory. Future observational research should adopt more robust designs, explicit confounding control, and transparent reporting to strengthen inferences about BTX‐A effectiveness in routine clinical practice.

Taken together, the RoB 2 and ROBINS‐I assessments indicate that the overall certainty of evidence regarding BTX‐A in PNP is limited. Many RCTs and most nonrandomized studies suffer from important risks of bias, small sample sizes, and short follow‐up. The synthesis in the subsequent sections therefore focuses not only on whether studies reported statistically significant improvements but also on how these findings align with study quality, consistency across designs, and the plausibility of observed effects.

### 3.2. Demographic and Etiological Characteristics: Age, Sex, Diagnosis, Treatment Periods, and Their Geographic Distribution

An analysis of the demographic and etiological features, treatment durations, and geographic distribution in these studies shows that BTX‐A has been tested in heterogeneous patient populations with a broad range of chronic pain disorders. The patient groups studied (1343 patients: 547 males and 632 females, where specified) show considerable diversity in both age and gender, indicating that BTX‐A has been applied across different populations rather than in a narrowly defined subgroup. For example, some research, such as the study on interstitial cystitis/bladder pain syndrome (IC/BPS), concentrated solely on female participants [73], reflecting the sex‐specific nature of the condition, whereas other studies included both male and female subjects, suggesting broader applicability across genders. The range of diagnoses addressed with BTX‐A is similarly broad, spanning NP arising from peripheral nerve injuries [81], CRPS [80, 81], residual or phantom limb discomfort [76], TN [93, 94], and temporomandibular joint disorder. The inclusion of an RCT that examined the effectiveness of BTX‐A in treating PLP further illustrates that its use has extended beyond craniofacial pain conditions [95]. Treatment protocols also showed substantial variation: Some studies used single injections with follow‐up durations of several weeks, others focused on repeated injections over several months, and a few explored long‐term effects for more than a year, underlining the absence of a uniform treatment schedule and the need to clarify optimal dosing strategies [91]. The geographic range of these studies is wide, covering Europe [10, 72, 85], Asia [74, 76], the Middle East [69, 71], and both North and South America [73, 79]. In addition, Brazilian research concentrated on 13 individuals with TN, whereas an American pilot study encompassed 44 patients with a broader spectrum of facial pain disorders, such as TN, temporomandibular joint syndrome, and postsurgical pain [93]. Dosage and administration methods were tailored to the clinical context, varying from transcutaneous injections in the Brazilian study to intradermal applications in other trials, illustrating how injection technique and dose were adapted to different target regions rather than standardized across studies [71, 95]. Importantly, the finding in the American study of an association between inflammatory markers and favorable treatment response suggests that a detailed understanding of underlying pathophysiology may be relevant for patient selection. Overall, the worldwide availability and diverse patterns of use of BTX‐A in these heterogeneous populations emphasize the need for additional, well‐designed studies to refine treatment methods and determine in which chronic pain disorders and patient subgroups BTX‐A can be beneficial.

### 3.3. Safety and Adverse Events

The safety profiles and documented adverse events in these studies suggest that BTX‐A is generally well tolerated, although a range of mostly mild and transient side effects has been reported. Reactions at the local injection site, including pain, hematoma, and swelling, were frequently noted and are likely related to the injection procedure itself [31, 68, 84]. Studies focusing on TN consistently observed temporary facial asymmetry, probably because of the proximity of injection sites to facial muscles, indicating that aesthetic side effects are a recurrent issue in facial treatments [76, 83, 90, 93, 94]. For example, Piovesan et al. [93] reported asymmetrical facial wrinkles in three individuals and mild eyelid ptosis in one, whereas Borodic and Acquadro [94] observed facial asymmetry and weakness. Neurological side effects were uncommon but included temporary facial weakness and dysesthesia, highlighting the possibility that BTX‐A may affect adjacent neural structures [68, 77]. Studies of BTX‐A for conditions such as IC/BPS identified urinary tract infections (UTIs) and short‐term urinary retention, underscoring potential genitourinary complications [73]. Conversely, some studies, especially those examining diabetic peripheral neuropathy (DPN), reported no adverse events related to BTX‐A injections [69]. Alizadeh et al. [95], in a double‐blind RCT evaluating BTX‐A for PLP, stated that side effects were monitored in both the BTX‐A and placebo groups but did not provide detailed information, making it difficult to fully characterize the safety profile in this context. Moreover, several studies with open‐label designs or limited adverse‐event documentation contributed only sparse information on safety [87–90]. One notable serious adverse event involving severe distress was reported in a study of chronic pelvic pain [85]. In summary, although BTX‐A appears to have a generally favorable short‐term tolerability profile, the heterogeneity of reporting, modest sample sizes, and relatively short follow‐up periods mean that uncommon or delayed adverse events cannot be excluded. It therefore remains essential to carefully weigh potential local and systemic side effects in relation to injection site, dosage, and individual patient factors when considering BTX‐A treatment.

#### 3.3.1. Effect Size and Certainty of Evidence

The effectiveness of BTX‐A in relieving different NP conditions has been investigated in multiple studies, which report a range of effect sizes and varying levels of confidence in the findings. In TN, several studies have shown significant decreases in pain intensity, typically assessed with the VAS, together with reductions in the frequency of episodes; in many of these reports, a considerable proportion of patients achieved at least a 50% reduction in pain intensity after BTX‐A treatment [76, 84]. Comparable benefits have been described in DPN, where significant improvements in VAS and NPS scores were observed over relatively short follow‐up intervals [69, 71]. However, these beneficial effects were not consistent across all pain dimensions in DPN, as some specific sensations, such as dull or cold feelings, appeared less responsive to BTX‐A therapy [71]. The certainty of the evidence supporting these observations is strongly influenced by the methodological rigor and design of the individual studies. Randomized, double‐blind, placebo‐controlled trials, particularly those with larger sample sizes and robust methodology, generally provide higher levels of certainty regarding BTX‐A’s efficacy [72, 74]. In contrast, studies using open‐label designs or enrolling small samples, including those evaluating BTX‐A for conditions such as carpal tunnel syndrome (CTS) and CRPS, are more prone to bias and offer lower‐certainty evidence [80, 88]. Crossover trials may help reduce inter‐patient variability but have inherent limitations, especially the risk of carryover effects between treatment phases, which can complicate interpretation of treatment differences [78]. Additional information on effect size and certainty comes from selected open‐label and controlled studies. For example, Piovesan et al. [93] reported notable reductions in VAS pain intensity across all three branches of the trigeminal nerve following BTX‐A injection, with the greatest effect seen 20 days after treatment (*p* < 0.05) and a concomitant reduction in analgesic use, including complete discontinuation of pain medication in four participants. Borodic and Acquadro [94] similarly observed that approximately 75% of patients with chronic facial pain responded to BTX‐A, defined as at least a 50% reduction in pain frequency or intensity or reduced reliance on analgesic therapy, and found a statistically significant association (*p* < 0.01) between inflammatory markers and a positive response to treatment. However, in both studies the open‐label design and absence of control groups limit the confidence that these large effect sizes can be generalized to wider populations. By contrast, Alizadeh et al. [95] conducted a double‐blind RCT in PLP and reported statistically significant decreases in VAS scores in the BTX‐A group compared with placebo at weeks 2, 4, and 8 (*p* < 0.05), providing a higher level of evidence for a possible treatment effect in this specific condition. Even in this trial, however, incomplete reporting of the exact magnitude of pain relief and limited details on adverse events constrain the overall certainty of the evidence. Taken together, these findings suggest that BTX‐A may yield clinically relevant pain reductions in selected NP conditions, but the strength of evidence ranges from moderate in better‐designed RCTs to low or very low in small, uncontrolled, or crossover studies, and the true effect size remains uncertain.

### 3.4. Efficacy of BTX Injections in Reducing Pain Intensity and Functional Outcomes

The available studies collectively suggest that BTX‐A injections can reduce pain and, in some cases, improve functional abilities in several chronic PNP conditions, although the strength and consistency of these effects vary across syndromes and study designs. In TN, multiple trials have reported statistically significant decreases in pain intensity and attack frequency after BTX‐A treatment across different dosages and injection techniques [84, 90]. Some studies have also described reductions in the need for concomitant analgesic medication, with a subset of patients becoming medication‐free following BTX‐A injections [92–94]. In a similar manner, intradermal BTX‐A injections have been associated with pain improvement in DPN, sometimes accompanied by additional benefits such as better sleep quality and fewer muscle cramps [31, 89]. Subcutaneous or intradermal BTX‐A has also been reported to reduce pain in PHN, with some studies noting concomitant improvements in sleep and reduced dependence on opioid drugs [10, 74, 75]. Beyond these more frequently investigated conditions, BTX‐A has been explored in other challenging NP syndromes, including general PNP [72] and chronic NP [10], but evidence in these areas remains limited and often comes from small or single‐center studies. BTX‐A has been reported to prolong the analgesic effect of sympathetic nerve blocks in CRPS [81] and to alleviate persistent residual limb pain in amputees [76], while a controlled trial in phantom pain showed a greater reduction in pain scores with BTX‐A compared with placebo [95]. Early BTX‐A treatment in synkinesis related to Bell’s palsy has been linked to functional improvements [86], and BTX‐A has demonstrated potential analgesic effects in occipital neuralgia [87]. Conversely, evidence for BTX‐A efficacy in CTS [79, 88] and chronic pelvic pain [85] is inconclusive or negative, particularly in higher‐quality randomized trials. All in all, these findings indicate that BTX‐A may offer clinically meaningful pain relief for selected PNP conditions, but the overall body of evidence is heterogeneous and generally based on small samples, short follow‐up, and studies with varying risk of bias. BTX‐A should therefore be regarded as a promising but still incompletely validated option, whose role and indications need to be further defined through larger, methodologically rigorous trials. Table S1 summarizes the main characteristics and outcomes of all studies included in this review.

## 4. Discussion

### 4.1. The Role of BTX‐A in Managing Pain: A Qualitative Analysis of Efficacy and Variability

This qualitative synthesis brings together results from randomized and nonrandomized studies on BTX‐A for PNP and situates them within the broader NP literature. Within this body of evidence, the most consistent and clinically meaningful signals of benefit are observed in prototypical, often treatment‐refractory PNP syndromes with a clearly defined peripheral nerve lesion, such as TN, DPN, PHN, PLP, post‐traumatic or postsurgical NP, and other focal peripheral nerve injuries [70, 74–76, 83, 87, 92, 93, 95]. In these core PNP indications, several RCTs and observational studies describe clinically relevant reductions in pain intensity and improvements in sleep or quality of life compared with placebo or usual care [70, 74–76, 83, 87, 92, 93]. Other neuropathic or neuropathic‐like conditions, including CRPS and CTS, show more inconsistent or negative findings, with isolated trials suggesting benefit and others failing to demonstrate superiority over placebo [79, 80, 82, 88]. These patterns indicate that BTX‐A cannot be considered a universal treatment for PNP and that its potential benefit is likely phenotype‐specific and influenced by injection protocol and baseline sensory profile. The interpretation of these efficacy signals should remain cautious because most studies are small, often single‐center, and affected by at least some concerns or a high risk of bias. In our qualitative synthesis, we therefore prioritized data from core PNP phenotypes and interpreted findings from other chronic pain conditions more cautiously. A smaller subset of included studies evaluated BTX‐A in nonclassical or mixed‐mechanism pain conditions in which neuropathic and nociceptive components coexist to varying degrees. In IC/BPS [73], the primary pathophysiology encompasses urothelial dysfunction and inflammatory and possibly autoimmune processes, but many patients report burning suprapubic pain, urinary urgency, and sensory hypersensitivity that are compatible with neuropathic‐like features and central sensitization. Similarly, in women with chronic pelvic pain and increased pelvic floor muscle tone [85], myofascial overactivity and nociceptive input from pelvic floor muscles are prominent, yet allodynia, hyperalgesia, and referred pain patterns suggest overlapping neuropathic mechanisms in at least a subgroup of patients. In the open‐label series on chronic facial pain [94], idiopathic trigeminal neuralgia, consistent with a classical neuropathic phenotype, was analyzed together with temporomandibular joint disorder, postsurgical facial pain, and chronic or essential headache, which are more typically nociceptive or mixed disorders. Finally, the retrospective study on facial synkinesis after Bell’s palsy [86] primarily addressed involuntary movements as a motor complication; pain was an associated but not the primary outcome, and any analgesic effect of BTX‐A is difficult to disentangle from improvements in abnormal muscle activity. These studies were retained because they report pain outcomes in clinical scenarios where neuropathic‐like symptoms and central sensitization are believed to contribute, but they cannot be regarded as pure models of PNP. In the present review, they are therefore considered as mixed nociceptive–neuropathic conditions, and their results are interpreted as exploratory and hypothesis‐generating. These nonclassical indications also illustrate the dynamic nature of the nociceptive–neuropathic continuum. Persistent inflammatory or myofascial nociceptive input in conditions such as IC/BPS, chronic pelvic pain, and some chronic facial pain syndromes may, over time, induce peripheral and central sensitization, small‐fiber dysfunction, and ectopic activity, leading to burning pain, allodynia, and hyperalgesia that overlap clinically with NP. We do not propose to reclassify these disorders as definite NP; rather, their inclusion reflects real‐world clinical practice, in which patients frequently present with mixed nociceptive–neuropathic phenotypes. In this framework, it is mechanistically plausible that BTX‐A, by inhibiting the release of pro‐algesic neuropeptides from C‐fiber terminals, reducing neurogenic inflammation, and indirectly modulating central sensitization, could provide benefit in selected mixed‐mechanism pain states, albeit with less predictable and less well‐validated effects than in core PNP syndromes.

Findings from this review align in part with the recent systematic review by Val and colleagues, which focused on orofacial NP disorders and concluded that BTX‐A can reduce pain intensity and improve quality of life in conditions such as trigeminal neuralgia and painful post‐traumatic trigeminal neuropathy, while highlighting substantial heterogeneity and the need for standardized protocols [36]. The present work extends those observations in two main ways. First, the scope includes a broader range of peripheral neuropathic syndromes beyond the orofacial region, allowing comparison of effect patterns across cranial and limb neuropathies. Second, the integration of RCTs and nonrandomized evidence, combined with formal risk‐of‐bias assessment, permits a more granular appraisal of how consistent and robust the apparent benefits are across different designs and clinical contexts. Both reviews converge on the notion that BTX‐A represents a potentially useful option in selected, localized NP syndromes but that current evidence does not yet support widespread adoption across all PNP indications.

Current guideline frameworks place BTX‐A as a later‐line option for NP rather than a first‐line therapy. NeuPSIG and related consensus statements recommend gabapentinoids, tricyclic antidepressants, and serotonin–noradrenaline reuptake inhibitors as initial pharmacologic choices, with topical agents and other systemic drugs as second‐line options, and consider BTX‐A only after failure or intolerance of these established treatments [35, 97]. Recent high‐level syntheses of pharmacotherapy and noninvasive neuromodulation for NP reached similar conclusions, emphasizing that evidence for BTX‐A is encouraging but still limited in scope and quality when compared with standard agents [35]. The present review supports this positioning: BTX‐A can be considered for highly selected patients with focal, refractory PNP in whom guideline‐recommended treatments have been exhausted or poorly tolerated, but available data do not justify routine use as a primary or broadly applied intervention.

These differences highlight that BTX‐A mechanisms probably focus on particular pathophysiological processes, and their efficacy is highly dependent on the underlying condition. Elements like the presence of allodynia might indicate better results in specific neuropathic conditions [10, 72]. Concerning practical use, different studies have utilized variable dosages (25 U–300 U) and methods of administration, suited to the condition, without consistently demonstrating a definitive dose–response relationship [68]. Significantly, multiple administrations seem advantageous, improving and prolonging analgesia for durations ranging from 24 weeks to 1 year [72, 91], thus indicating a potential for maintenance therapy. The length of effect usually lasts for a minimum of 8–12 weeks, occasionally lowering the necessity for retreatment in comparison to other options [73]. Assessing the evidence rigorously involves detecting both the methodological advantages and drawbacks. The trust in BTX‐A effectiveness for TN, DPN, PHN, and peripheral nerve injuries is largely based on several well‐structured, double‐blind RCTs (mentioned earlier). The RCT by Alizadeh et al. [95] offers comparably high‐quality evidence for PLP. Nevertheless, part of the literature, especially that concerning earlier studies or those exploring wider categories such as chronic facial pain, depends on open‐label designs lacking control groups [77, 87]. Although these studies offer useful initial indications, their findings are constrained by the difficulty in ruling out placebo effects or natural variations in the disease [92–94]. Additionally, constraints like limited sample sizes in preliminary studies [81, 88], the risk of unblinding from apparent side effects such as facial asymmetry [84], and variation in methodologies across research require careful interpretation and underscore the continuing demand for thorough research, especially well‐controlled RCTs for conditions presently backed predominantly by lower‐level evidence. In most studies, BTX‐A shows a positive safety profile. Adverse events are usually mild, temporary, and localized, most frequently involving injection site discomfort [10, 31, 74, 75] and localized muscle weakness, which can manifest as facial asymmetry or slight ptosis after injections for TN or facial pain [68, 76]. Larger doses might elevate the likelihood of weakness [68, 79]. The overall lack of systemic side effects presents a favorable comparison to numerous traditional oral analgesic drugs [72]. Nonetheless, certain situations require careful consideration, like the low tolerability noted in CRPS [80] or the potential for UTI and urinary retention associated with intradetrusor injections [73]. The present review identifies BTX‐A as an effective and usually safe treatment choice for various difficult, frequently resistant pain disorders, particularly certain NP syndromes such as TN, DPN, PHN, and possibly PLP. Its clinical effectiveness is evidenced by substantial pain alleviation and improvement in function and quality of life, noted in various studies, especially strong RCTs. However, its effectiveness relies on specific conditions, emphasizing the importance of thorough patient evaluation and diagnosis. All in all we emphasize that the strongest inferences about BTX‐A in NP derive from well‐characterized PNP syndromes with clear nerve injury, whereas data from IC/BPS [73], chronic pelvic pain with pelvic floor hypertonicity [85], heterogeneous chronic facial pain [94], and Bell’s palsy‐related synkinesis [86] should be viewed as illustrative of BTX‐A’s broader analgesic potential but insufficient to support firm conclusions regarding classical PNP.

### 4.2. Exploring the Neurophysiological Effects of BTX‐A in Animal Models: Implications for Pain Management and Beyond

The therapeutic mechanisms of BTX‐A are broad and encompass motor and sensory pathways, although clinical studies indicate the efficacy of botulinum neurotoxin in numerous pain states [98, 99]. From a neurophysiological perspective, BTX‐A can exert its effects by interfering with neurotransmitter release from nerve terminals by cleaving soluble N‐ethylmaleimide‐sensitive factor attachment protein (SNARE) proteins, more specifically, during the fusion of synaptic vesicles, synaptosomal‐associated protein 25 (SNAP‐25) [100–102]. Its action was initially reported to act at the NMJ, where inhibiting ACh release from nerve terminals prevents muscle fiber contraction [103]. This targeted muscle relaxation, or chemodenervation, is fundamental to its established efficacy in treating spasticity, directly reducing the muscle hypertonia and involuntary contractions associated with upper motor neuron syndromes [104]. While this neuromuscular blockade addresses motor symptoms and can indirectly alleviate pain associated with muscle overactivity, a possible BTX‐A influence on the sensory system involved in pain processing has been hypothesized [105]. In these sensory pathways, BTX‐A has also been shown to block the release of important pro‐algesic mediators such as CGRP and substance P at peripheral sensory nerve endings in animal models of inflammatory pain (formalin or complete Freund’s adjuvant injection), also reducing nocifensive behaviors [106, 107]. This inhibition of mediator release attenuates peripheral sensitization and neurogenic inflammation, thereby reducing the nociceptive input to the central nervous system and potentially modulating central sensitization, although direct central effects remain an active area of investigation [108]. Animal models help clarify these actions. In NP models of chronic constriction injury (CCI) or spared nerve injury (SNI), BTX‐A injections reduce mechanical allodynia and thermal hyperalgesia, in parallel with decreased CGRP/substance P expression in dorsal root ganglia (DRG) and reduced activation of spinal glial cells [109–111]. These findings underscore the role of BTX‐A in both sensory signaling and neuroinflammation. Studies utilizing models of TN have shown BTX‐A to inhibit neuronal hyper‐excitability in the trigeminal nucleus caudalis, but another study implies interference with transient receptor potential vanilloid 1 (TRPV1) function on sensory neurons [112–114]. Hence, studies in animals strongly demonstrate that BTX‐A can regulate not only motor hyperexcitability but also different aspects of nociceptive processing involved in pain transmission and perception, spanning peripheral neurotransmitter release and neurogenic inflammation to central neuronal activation [115–118]. Appreciating the different yet complementary actions by which BTX‐A exerts these neurophysiological actions is critical to better utilize BTX‐A therapeutically in its varying clinical applications in the fields of movement disorders and pain.

### 4.3. Charting the Future of BTX‐A in Pain Management: From Predictors to Synergistic Therapies

Building upon this foundation of clinical evidence and mechanistic insight, several key avenues for future research emerge that could further refine the therapeutic application of BTX‐A in pain management and beyond. Given the observed variability in treatment response across different pain conditions and even within diagnostic categories [79, 80, 85, 94], a critical future direction involves identifying robust predictors of efficacy. Research integrating detailed patient phenotypes, potentially including quantitative sensory testing, genetic analyses, or targeted biomarkers related to neuroinflammation or specific neuropeptide activity (like CGRP levels), could help delineate patient subgroups most likely to benefit, thereby enabling more personalized treatment strategies. Such studies are essential to move beyond the current trial‐and‐error approach often employed when standard therapies fail, particularly for conditions where BTX‐A efficacy is less consistently established than in TN or DPN [68–70]. Furthermore, while the peripheral mechanisms involving inhibition of neurotransmitter release from nociceptors are increasingly well understood [105–107], the precise contribution of potential central effects warrants more profound investigation. Elucidating the extent and functional consequences of retrograde axonal transport and subsequent modulation of central sensory processing, including spinal glial activation [109–111], could reveal additional therapeutic targets or explain long‐lasting effects observed clinically. Advanced neuroimaging techniques and refined electrophysiological studies in both preclinical models and human subjects could provide valuable insights into these central modulatory actions. Concurrently, optimizing treatment protocols remains a priority. Future research should focus on comparative effectiveness studies evaluating different dosages, injection techniques (e.g., image‐guided injections and varying depths based on target tissue), and retreatment intervals tailored to specific pain conditions and guided by mechanistic understanding [68, 72, 91]. Establishing evidence‐based guidelines for these parameters would enhance consistency and potentially improve long‐term outcomes.

### 4.4. Clinical Implications: Patient Selection, Injection Strategies, and Expected Benefit

The evidence summarized in this review suggests that BTX‐A should be reserved for patients with well‐characterized, focal PNP who remain symptomatic despite adequate trials of first‐ and second‐line pharmacologic therapies. Suitable candidates typically present with localized positive sensory symptoms, such as spontaneous burning pain, allodynia, or hyperalgesia in a relatively restricted territory, and without major motor impairment that could be exacerbated by chemodenervation. Clinical experience from the included studies indicates that conditions such as classical trigeminal neuralgia, painful DN with distal allodynia, and PHN with stable dermatomal pain are more likely to respond than widespread or poorly localized pain states [10, 31, 70, 74–76, 87, 92, 93].

Dosing and injection protocols were variable across trials, yet some pragmatic patterns emerged. Cranial and orofacial NP was usually treated with total doses between 25 and 100 units of BTX‐A distributed in small aliquots across the painful area or along branches of the trigeminal nerve, whereas limb neuropathies and postamputation pain often required higher total doses, typically between 50 and 300 units, administered intradermally or subcutaneously in a grid‐like pattern over the symptomatic region [10, 31, 75, 82, 92, 93]. Most studies reported onset of analgesic effect within one to two weeks, peak response around four weeks, and duration of benefit between eight and twelve weeks, after which retreatment was considered. Clinicians should start with conservative doses within the ranges supported by the trials, monitor carefully for local weakness or unwanted sensory changes, and individualize retreatment intervals based on response and tolerability.

Safety data from the included studies indicate that BTX‐A injections are generally well tolerated when administered by experienced clinicians. The most frequent adverse events were mild and transient, including local pain, bruising, or short‐lived facial asymmetry in cranial applications. Serious adverse events were rare but cannot be ruled out because of small sample sizes and incomplete reporting. Particular attention is warranted when injections are performed near motor branches or in patients with neuromuscular comorbidities. Shared decision‐making is therefore essential, and patients should be informed that BTX‐A is used off‐label for many PNP indications, with evidence that is promising yet still evolving.

### 4.5. Strengths and Limitations

This systematic review provides a methodologically rigorous synthesis of available evidence on the efficacy and safety of BTX‐A for PNP, an area of high clinical relevance given the often intractable nature of these conditions and the limitations of current treatment options. We used a comprehensive search strategy across multiple major databases (PubMed, Web of Science, Cochrane Library, Embase, EBSCOhost, and Scopus), with sensitive, PICO‐informed keywords and MeSH terminology, and did not restrict the search by publication date. We also applied established risk‐of‐bias tools (Cochrane RoB 2 for RCTs and ROBINS‐I for nonrandomized studies), which allowed systematic appraisal of the reliability and limitations of the included trials.

However, several limitations need to be considered. Inclusion of diverse study designs (from RCTs to observational studies) increases clinical generalizability but introduces substantial heterogeneity, which precludes robust meta‐analysis and requires cautious interpretation of apparent efficacy signals. Our restriction to full‐text articles in English may have introduced language bias and led to omission of relevant studies published in other languages. Moreover, the generalizability of the findings is constrained by the demographic and healthcare settings of the included populations, which are not uniformly representative of global practice. Although two recent systematic reviews and meta‐analyses [34, 35] have addressed related questions, our work focuses specifically on BTX‐A, with emphasis on mechanisms of analgesia and on off‐label use across multiple PNP phenotypes (TN, DPN, PHN, CRPS, PLP, and others). By combining data from different etiologies and designs and adopting a structured narrative synthesis rather than a formal meta‐analysis, we aimed to provide a clearer overview while acknowledging that pooling across highly heterogeneous protocols could yield misleading summary estimates. The SWiM‐informed approach used here [63] improves transparency about grouping decisions and effect directions, but it cannot fully compensate for the limitations of the underlying primary studies. Readers should therefore interpret patterns of benefit and harm in light of these constraints and avoid extrapolating beyond the clinical contexts that have actually been studied. An additional, conceptually important limitation concerns the clinical spectrum of the included conditions. Alongside prototypical PNP syndromes, such as TN, DPN, PHN, PLP, and focal peripheral nerve injuries, we deliberately retained studies in which the primary diagnosis is not a classical PNP disorder, including IC/BPS [73], chronic pelvic pain with pelvic floor muscle hypertonicity [85], heterogeneous chronic facial pain syndromes [94], and Bell’s palsy‐related facial synkinesis [86]. In these settings, pathophysiological mechanisms are likely mixed or predominantly nociceptive and motor, with neuropathic‐like features and central sensitization believed to contribute in at least a subset of patients. Their inclusion was justified because they report pain outcomes in clinically relevant scenarios where nociceptive and neuropathic mechanisms coexist and where BTX‐A is increasingly used in practice; however, it inevitably increases clinical and mechanistic heterogeneity and limits the extent to which our conclusions can be interpreted as pertaining to “pure” PNP. Accordingly, the conclusions of this review should be considered most robust for clearly defined PNP phenotypes (e.g., TN, DPN, PHN, and PLP), whereas findings in IC/BPS, chronic pelvic pain, chronic facial pain, and synkinesis should be regarded as exploratory and hypothesis‐generating rather than definitive evidence for NP indications.

To address this issue analytically, the narrative synthesis conceptually separated prototypical PNP indications from these mixed‐mechanism conditions and placed greater interpretative weight on high‐quality RCTs in classical PNP when formulating the main clinical implications. In particular, effect estimates from heterogeneous cohorts (such as chronic facial pain [94]) and from studies primarily targeting motor complications (such as facial synkinesis after Bell’s palsy [86]) were not treated as equivalent to data from trials in well‐characterized neuropathic syndromes. The present review does not claim that BTX‐A is broadly effective across all chronic pain syndromes; rather, it highlights selective benefit in specific neuropathic phenotypes, with more uncertain and context‐dependent effects in complex mixed nociceptive–neuropathic disorders.

## 5. Conclusions

In conclusion, this systematic review indicates that BTX‐A may provide clinically relevant pain relief for selected PNP conditions, most consistently trigeminal neuralgia, painful DN, and PHN. Evidence from randomized trials and observational studies suggests improvements in pain intensity and related outcomes for some patients, with a generally favorable short‐term safety profile. The overall certainty of evidence remains low to moderate, however, because many studies are small, single‐center, and affected by important risks of bias and heterogeneous protocols. Convincing efficacy has not been demonstrated for several other PNP syndromes, including CRPS, CTS, and chronic pelvic pain, where results are negative or inconsistent. Clinical use of BTX‐A in PNP should therefore remain cautious and individualized, ideally restricted to patients with focal, refractory pain who have not responded to guideline‐recommended first‐ and second‐line treatments and who are managed within multidisciplinary pathways. Further high‐quality, adequately powered RCTs with standardized outcome measures, longer follow‐up, and rigorous reporting of adverse events are required to clarify the true magnitude and durability of benefit, to refine injection strategies, and to better define the place of BTX‐A within NP treatment algorithms.

**Nomenclature**
ACh: Acetylcholine
BTX: Botulinum toxin
BTX‐A: Botulinum toxin A
CCI: Chronic constriction injury
CGRP: Calcitonin gene‐related peptide
CRD: Registration prefix used by PROSPERO
CRPS: Complex regional pain syndrome
CTS: Carpal tunnel syndrome
DPN: Diabetic peripheral neuropathy
DRG: Dorsal root ganglia
EFNS: European Federation of Neurological Societies
IC/BPS: Interstitial cystitis/bladder pain syndrome
NMJ: Neuromuscular junction
NP: Neuropathic pain
NPS: Neuropathy Pain Scale
PGIC: Patient global impression of change
PHN: Postherpetic neuralgia
PLP: Phantom limb pain
PNP: Peripheral neuropathic pain
PRISMA: Preferred Reporting Items for Systematic Reviews and Meta‐Analyses
PROSPERO: Name of a registration database
PSQI: Pittsburgh sleep quality index
RCTs: Randomized controlled trials
RoB: Risk of bias
ROBINS‐I: Risk of Bias in Nonrandomized Studies – of Interventions
SF‐36: Short form‐36
SNARE: Soluble N‐ethylmaleimide‐sensitive factor attachment protein receptor
SNI: Spared nerve injury
SNAP‐25: Synaptosomal‐associated protein 25
TN: Trigeminal neuralgia
TRPV1: Transient receptor potential vanilloid 1
UTIs: Urinary tract infections
VAS: Visual analog scale

## Ethics Statement

As this systematic review involves secondary data analysis from previously published studies, no new ethical approval was required.

## Consent

The authors have nothing to report.

## Disclosure

All authors have read and agreed to the published version of the manuscript.

## Conflicts of Interest

The authors declare no conflicts of interest.

## Author Contributions

Conceptualization: Domenico Antonio Restivo, Andrea Calderone, Rocco Salvatore Calabrò, and Antongiulio Bruschetta; methodology: Domenico Antonio Restivo, Andrea Calderone, Rocco Salvatore Calabrò, and Antongiulio Bruschetta; validation: Domenico Antonio Restivo, Rocco Salvatore Calabrò, and Antongiulio Bruschetta; investigation: Domenico Antonio Restivo, Andrea Calderone, and Antongiulio Bruschetta; resources: Rocco Salvatore Calabrò; data curation: Domenico Antonio Restivo and Andrea Calderone; writing–original draft preparation: Domenico Antonio Restivo, Andrea Calderone, Angelo Quartarone, and Rocco Salvatore Calabrò; writing–review and editing: Domenico Antonio Restivo, Andrea Calderone, Angelo Quartarone, and Rocco Salvatore Calabrò; visualization: Andrea Calderone; supervision: project administration; and funding acquisition: Angelo Quartarone and Rocco Salvatore Calabrò. Rocco Salvatore Calabrò and Antongiulio Bruschetta have equally contributed and co‐last authors to the work.

## Funding

This study was supported by Current Research Funds 2025, Ministry of Health, Italy. RRC‐2025‐23686388.

## Supporting Information

Table S1: Summary of the included studies.

## Supporting information


**Supporting Information** Additional supporting information can be found online in the Supporting Information section.

## Data Availability

Data sharing is not applicable to this article, as no datasets were generated or analyzed during the current study.
