# Supplementary material for: The Use of Botulinum Toxin Injections in Peripheral Neuropathic Pain: A Systematic Review of Efficacy and Safety Outcomes
Source: Pain Res Manag. 2026 Jan 16;2026:7701940. doi: 10.1155/prm/7701940 (PMC12811149; doi:10.1155/prm/7701940)
Supplement: Supplementary file 1 — Supporting Information Additional supporting information can be found online in the Supporting Information section. [file PRM-2026-7701940-s001.docx]

**Table 3. Summary of the included studies.**

| **Author/Location/**  **Country of the Study** | **Aim** | **Study Design/Sample Size/Demographic Data/Diagnosis/Treatment Period** | **Outcomes Measures and Adverse Events** | **Main Findings** | **Effect Size/Certainty of Evidence** | **Dosage/Injection of Botulinum Toxic and Efficacy** |
| --- | --- | --- | --- | --- | --- | --- |
| Zhang et al. 2014 [68]  **Location:** First Affiliated Hospital of Zhengzhou University **Country:** China | To assess the effectiveness and safety of BTX-A in treating patients with TN. | **Study Design:** RCT.  **Size:** 84 patients were randomized (80 patients were included in the final analysis). Placebo: 28; BTX-A 25U: 27; BTX-A 75U: 29. **Age:** Mean age 58-62 years across groups. **Sex:** Placebo: 51.85% male; BTX-A 25U: 40% male; BTX-A 75U: 42.86% male  **Diagnosis:** TN according to ICHD-II criteria.  **Treatment Period:** 9 weeks (1-week baseline, 8-week follow-up). | VAS for pain severity. PGIC scale. Proportion of responders (≥ 50% reduction in VAS). Adverse event recording.  Mild to moderate adverse events: facial asymmetry, transient edema. All adverse events resolved within weeks. No serious adverse events. | Both the 25U and 75U BTX-A groups showed a notable decrease in pain compared to the placebo group. Nonetheless, no notable difference in effectiveness was noted between the two dosage groups. Moreover, a greater proportion of patients in both the 25U and 75U groups were identified as responders in comparison to those in the placebo group. Additionally, a higher percentage of patients in these categories indicated a "much improved" or "very much improved" rating on the PGIC scale compared to the placebo group. | **Effect Size:** VAS scores: P < 0.017 (BTX-A vs placebo). Responder rate: P < 0.017 (BTX-A vs placebo). PGIC: P < 0.017 (BTX-A vs placebo). No significant differences between the 25U and 75U groups (P > 0.05).  **Certainty of Evidence:** Moderate. This is because it is a RCT, but with a small sample size. | **Dosage/Injection BTX:** 25U and 75U total doses of BTX-A. Injections administered intradermally and/or submucosally. 20 injection points, 0.05ml per point.  **Efficacy of BTX:** Effective in reducing pain in TN. No significant difference in efficacy between 25U and 75U doses. Rapid pain relief was observed within the first week. |
| Ghasem et al. 2014 [69]  **Location:** Al-Zahra and Kashani Hospitals, Isfahan **Country:** Iran | To assess the impact of intradermal BTX-A injections on neuropathic pain in individuals with type 2 diabetes. | **Study Design:** RCT.  **Size:** 40 patients (20 in each group). **Age:** Mean age 59-62 years across groups. **Sex:** Placebo: 9 male, 11 female. BTX-A: 13 male, 7 female.  **Diagnosis:** DN diagnosed using the DN4 questionnaire and nerve conduction velocity examinations.  **Treatment Period:** 3 weeks. | NPS, VAS, DN4, nerve conduction velocity examinations.  No adverse events were reported in either group. | The BTX-A group showed marked enhancement in NPS scores relative to the placebo group. Moreover, individuals in the BTX-A group saw a significant decrease in pain severity, as assessed by the VAS. Moreover, a notable decline was noted in certain items of the DN4 questionnaire, reflecting a general decrease in neuropathic pain symptoms within the BTX-A group. | **Effect Size: VAS:** P = 0.01 (BTX-A vs. placebo). NPS: P < 0.001 for several NPS items (BTX-A vs. placebo). DN4: P<0.05 for electric shocks, burning, pins and needles and brushing in the BTX-A group.  **Certainty of Evidence:** Moderate. The study is a RCT, which is a good study design, but the small sample size limits the certainty of the evidence. | **Dosage/Injection BTX:** 100 units of BTX-A (Dysport) administered intradermally. 12 injection sites (8-10 units per site) on the dorsum of one foot.  **Efficacy of BTX:** BTX-A was effective in reducing neuropathic pain in diabetic patients, as demonstrated by significant improvements in NPS and VAS scores. |
| Salehi et al. 2019 [70]  **Location:** Ali Ibn Abi Talib Hospital, Rafsanjan **Country:** Iran | To examine the effectiveness of BTX-A injections in alleviating neuropathic pain and enhancing quality of life and sleep in individuals with DSPN. | **Study Design:** RCT.  **Size:** 32 patients (16 in each group). **Age:** Mean age 56-58 years across groups. **Sex:** Intervention Group: 6 male, 10 female. Placebo Group: 6 male, 10 female.  **Diagnosis:** DSPN in type 2 diabetic patients, confirmed using the DN4 questionnaire and nerve conduction studies.  **Treatment Period:** 12 weeks (follow-up at 1, 4, 8, and 12 weeks post-injection). | VAS for pain intensity. NPS for pain quality and location. SF-36 for quality of life. PSQI for sleep quality.  The study did not explicitly report on adverse events, but implied good tolerability. | The BTX-A group showed a notable decrease in VAS scores when compared to the placebo group, suggesting effective pain alleviation. Moreover, individuals in the BTX-A cohort showed a significant increase in PSQI scores, indicating better sleep quality. Furthermore, a notable improvement was seen in the physical dimension of the SF-36 survey, indicating a beneficial effect on the overall quality of life. Additionally, the BTX-A group demonstrated notable enhancements in multiple NPS subscales, emphasizing positive alterations in the perception of pain quality. | **Effect Size:** VAS: P < 0.001 (BTX-A vs. placebo). PSQI: P < 0.001 (BTX-A vs. placebo). SF-36 (physical component): P < 0.001 (BTX-A vs. placebo). NPS subscales: P < 0.001 for several subscales (BTX-A vs. placebo). SF-36 for quality of life. PSQI for sleep quality.  **Certainty of Evidence:** Moderate. This is a double blind RCT, which provides good evidence, but the sample size is relatively small. | **Dosage/Injection BTX:** 100 units of BTX-A (Dysport) dissolved in 1.2 ml normal saline. 12 intradermal injection sites (8.33 units per site) on the foot.  **Efficacy of BTX:** BTX-A was effective in reducing neuropathic pain, improving sleep quality, and enhancing the physical component of quality of life in patients with DSPN. |
| Taheri et al. 2020 [71]  **Location:** Imam Hossein Medical Center, Tehran **Country:** Iran | To assess the efficacy of BTX-A injections in managing DSPN by measuring pain alleviation and modifications in symptoms related to neuropathy. | **Study Design:** RCT.  **Size:** 141 patients. Group D1 (BTX-A one foot, saline other foot): 47 patients. Group D2 (BTX-A both feet): 47 patients. Group N (saline both feet): 47 patients. **Age:** Mean age 54-57 years, with a range of 38-72 years. **Sex:** 55 (39.0%) males; 16 (34.0%) females.  **Diagnosis:** DSPN, confirmed using the DN4 questionnaire and nerve conduction velocity testing.  **Treatment Period:** Three months, with follow-up at 4 weeks post-injection. | VAS for pain intensity. NPS for pain quality and characteristics.  The research did not explicitly describe adverse events, yet suggested that the therapy was generally well-accepted. | Both BTX-A groups (D1 and D2) showed considerable pain relief, based on the VAS, and enhancements in most NPS parameters compared to the saline group (N). Nonetheless, there was no notable enhancement in dull and cold sensations after receiving BTX-A treatment. Notably, individuals who underwent BTX-A injections in one foot reported less pain in both feet, indicating a potential systemic or central modulation effect from the treatment. | **Effect Size:** VAS: P < 0.001 (BTX-A groups vs. saline group). NPS (various parameters): P < 0.001 for several parameters (BTX-A groups vs. saline group). Hot sensation D1 vs D2 P=0.003  **Certainty of Evidence:** Moderate. The study is a prospective RCT, which is a good study design. The large sample size is also a positive aspect. However, the study only followed the patients for 3 months, so long term effects are unknown. | **Dosage/Injection BTX:** 150 units of BTX-A total per patient. Group D1: 75 units in one foot, saline in the other. Group D2: 75 units per foot, total of 150 units. 20 intradermal injection points per foot (7.5 units per point in D1, and 3.75 units per point in D2).  **Efficacy of BTX:** BTX-A was effective in significantly reducing pain and improving several neuropathy-related symptoms in patients with DSPN, with the exception of dull and cold sensations. |
| Attal et al. 2016 [72]  **Location:** Clinical Pain Centre, Ambroise Paré Hospital. **Country:** France and Brazil (multicenter study) | To assess the effectiveness and safety of repeated subcutaneous injections of BTX-A versus placebo in individuals with peripheral neuropathic pain. | **Study Design:** RCT.  **Size:** 68 patients randomized (34 in the BTX-A group, 32 in the placebo group). 66 patients included in the Intention to treat analysis. **Age:** Mean age 51-52 years. **Sex:** BTX-A: 17 male, 17 female. Placebo: 20 male, 12 female.  **Diagnosis:** Peripheral neuropathic pain.  **Treatment Period:** 24 weeks, with two administrations of BTX-A or placebo 12 weeks apart. | The study evaluated changes in weekly BPI-NRS pain intensity as the primary outcome. Secondary measures covered safety, response rates, sensory and neuropathic symptoms, psychological status, quality of life, sleep, and skin biopsy markers. Findings focused on efficacy and suggested good tolerability, with injection-related pain as the main adverse effect. | BTX-A produced a reduction in average pain intensity compared with placebo and showed additional benefit after a second administration. Significant improvements were also observed in paroxysmal pain, allodynia, and related neuropathic symptoms, with higher baseline IENFD potentially predicting treatment response. Modest reductions in anxiety and better sleep quality further supported a broader improvement in well-being. | **Effect Size:** Effect sizes were robust across outcomes (primary pain p<0.0001; paroxysmal pain p<0.0001; allodynia p=0.0002; anxiety p=0.02; sleep p=0.02–0.03).  **Certainty of Evidence:** moderate to high, supported by a multicenter, double-blind RCT design. | **Dosage/Injection BTX:** Up to 300 units of BTX-A per administration. Subcutaneous injections at sites 1.5-2 cm apart (5 units per site). Two administrations 12 weeks apart.  **Efficacy of BTX:** BTX-A exhibited notable effectiveness in alleviating neuropathic pain, especially in individuals with allodynia, and indicated therapeutic benefits with repeated doses. It additionally enhanced particular neuropathic symptoms and resulted in moderate improvements in anxiety and sleep quality. |
| Welch et al. 2024 [73]  **Location:** single academic institution **Country:** United States | To compare the effectiveness of BIT and intradetrusor onabotulinumtoxinA injections for the treatment of IC/BPS. | **Study Design:** RCT.  **Size:** 58 patients enrolled, 47 included in the intention to treat analysis. (22 in the BIT group, 25 in the onabotulinumtoxinA group). **Age:** 18 years or older.  **Sex:** All female subjects.  **Diagnosis:** IC/BPS.  **Treatment Period:** 2 months post-treatment for primary outcomes, with follow-up at 6-9 months for secondary outcomes. | Primary: OLS questionnaire scores. Secondary: FSFI, FSDS-R, SF-12, VAS, patient satisfaction, retreatment rates, and adverse events.  UTIs occurred in 6 patients in each group. Urinary retention requiring clean intermittent catheterization occurred in 2 patients in the onabotulinumtoxinA group. One patient in the BIT group experienced self-limited side effects. | Intradetrusor onabotulinumtoxinA injection resulted in a markedly better symptom relief, indicated by reduced OLS values, at two months following treatment when compared to BIT. Nonetheless, this disparity was not maintained at the 6–9 month follow-up. Significantly, patients treated with onabotulinumtoxinA were less prone to needing retreatment during this time, indicating a prolonged therapeutic effect. In spite of these advantages, no notable differences were seen between the two groups concerning sexual function, mental health, or physical health outcomes. | **Effect Size:** OLS scores at 2 months: ICSI (P = 0.008), ICPI (P = 0.048). Retreatment rates: P = 0.0002.  **Certainty of Evidence:** Moderate. The randomized design is a strength, but the study was underpowered, and blinding was not possible. | **Dosage/Injection BTX:** 100 units of onabotulinumtoxinA reconstituted in 10 mL of normal saline. 0.5 mL injections at 20 sites along the posterior bladder wall.  **Efficacy of BTX:** Intradetrusor onabotulinumtoxinA injection was more effective than BIT in reducing IC/BPS symptoms at 2 months post-treatment and resulted in lower retreatment rates. |
| Xiao et al. 2010 [74]  **Location:** Shenzhen Nanshan Hospital, Guangdong Medical School, Shenzhen **Country:** China | To investigate the efficacy of subcutaneous BTX-A injection in alleviating the symptoms of PHN. | **Study Design:** RCT.  **Size:** 60 subjects enrolled, 56 completed the study. (20 in each of the placebo and lidocaine groups, and 16 in the BTX-A group). **Age:** Mean 68 ± 12.2 years (range 42-84 years). **Sex:** 28 males and 32 females.  **Diagnosis:** PHN  **Treatment Period:** 3 months. | VAS for pain intensity. Quality of life assessment (sleep time, daily activity, diet, stance). Percent of opioid use.  Pain during subcutaneous injection was reported. No serious adverse effects were reported. No allergic reactions were observed. | BTX-A showed considerable pain relief when compared to both lidocaine and placebo at 7 days and 3 months after treatment, emphasizing its long-lasting analgesic properties. Moreover, BTX-A resulted in a significant enhancement in sleep duration compared to the other groups, indicating additional advantages beyond just pain relief. Additionally, patients administered BTX-A showed a notable decrease in opioid consumption, suggesting its capability to lessen dependence on pain relievers. In comparison, although lidocaine offered pain relief on the first day, its effects did not last beyond this early stage. | **Effect Size:** Pain reduction (BTX-A vs. lidocaine/placebo): P < 0.01. Sleep time improvement (BTX-A vs. lidocaine/placebo): P < 0.01. Opioid use reduction (BTX-A vs. lidocaine/placebo): P < 0.01. Lidocaine pain reduction at day 1 vs the other groups P<0.01.  **Certainty of Evidence:** Moderate. The randomized, double-blind design is a strength. However, the dropout rate, and the use of a non standard BTX-A mixture, reduces the certainty of the evidence. | **Dosage/Injection BTX:** 100 IU/vial of BTX-A reconstituted with 20 mL of saline (5 u/mL). Subcutaneous injections into the affected area with tactile allodynia. Volumes of administration varied according to the area of tactile allodynia, but less than 40-mL volumes (200 units for the maximum BTX-A dose) were used.  **Efficacy of BTX:** BTX-A was effective in significantly reducing PHN pain, improving quality of life, and reducing opioid use compared to lidocaine and placebo. |
| Apalla et al. 2013 [75]  **Location:** First Department of Dermatology, Aristotle University of Thessaloniki **Country:** Greece. | To evaluate the effectiveness, safety, and tolerance of BTX-A against a placebo in managing PHN. | **Study Design:** RCT.  **Size:** 30 patients enrolled, 26 included in the analysis (15 in each group). **Age:** Mean age was 73.2 ± 10.5 years in the BTX-A group and 77.5 ± 8.2 years in the placebo group. **Sex:** BTX-A group: 8 males, 7 females. Placebo group: 10 males, 5 females.  **Diagnosis:** PHN with a VAS score ≥7 at baseline.  **Treatment Period:** 4 weeks, followed by an open-label 20-week follow-up phase for complete responders. | Primary: Reduction in VAS score within a 4-week period.  Secondary: Reduction in sleep score, maintenance of VAS score. safety and tolerability.  Pain during injections was reported in both groups. No other local or systemic side effects were recorded. No patients had to discontinue treatment due to discomfort. | BTX-A resulted in a notably larger decrease in pain intensity, as assessed by Visual Analog Scale (VAS) scores, in comparison to placebo, showcasing its effectiveness in pain treatment. Moreover, BTX-A notably enhanced sleep quality, further fostering patient wellness. The median time to reach a pain decrease exceeding 50% was 7.44 days, suggesting a fairly quick onset of effect. Additionally, the median time for pain relief was 16 weeks, indicating a lasting therapeutic impact. | **Effect Size:** VAS score reduction (BTX-A vs. placebo): P < 0.001. Sleep score reduction (BTX-A vs placebo) P<0.001; Cohen’s d effect size for VAS score reduction: 3.6 (week 2), 4.2 (week 4). Cohen's d effect size for sleep score reduction: 2.2 (week 2), 2.4 (week 4).  **Certainty of Evidence:** Moderate. The randomized, double-blind design is a strength. However, the relatively small sample size may limit the generalizability of the findings. | **Dosage/Injection BTX:** 100 IU of BTX-A (5 U/route) diluted with 4 mL of sodium chloride (0.9%). Subcutaneous injections in a chessboard manner over the affected area (40 injections total).  **Efficacy of BTX:** BTX-A was effective in significantly reducing pain and improving sleep quality in patients with PHN compared to placebo. |
| Ranoux et al. 2008 [10]  **Location:** Ambroise Pare´ Hospital, Boulogne-Billancourt **Country:** France | To examine the possible direct pain-relieving effects of BTX-A in individuals with localized neuropathic pain. | **Study Design:** RCT.  **Size:** 29 patients. BTX-A group: 15 patients. Placebo group: 14 patients. **Age:** Mean age was 53.8 ± 13.9 years in the BTX-A group, and 49.7 ± 15.9 years in the placebo group. **Sex:** 19 women, 10 men.  **Diagnosis:** PHN or posttraumatic/postoperative neuropathies with mechanical allodynia.  **Treatment Period:** 24 weeks. | Primary: Self-reported average pain intensity (11-point numerical scale). Secondary: Sensory deficits, pain thresholds, allodynia, neuropathic symptoms, quality of life, mood.  Pain during injections was reported in both groups. Mild side effects associated with the anesthetic procedure were reported. No serious adverse events were reported. | BTX-A led to a notable enhancement in weekly average pain levels when compared with placebo, highlighting its effectiveness in controlling chronic pain. Moreover, BTX-A significantly diminished allodynia and raised cold pain thresholds, indicating a change in sensory processing. In addition to alleviating pain, BTX-A also resulted in significant enhancements in neuropathic symptoms and overall life quality, emphasizing its wider therapeutic advantages for individuals suffering from neuropathic pain. | **Effect Size:** Pain improvement (BTX-A vs. placebo): p = 0.038 (LOCF analysis). Pain improvement at week 2 p = 0.025, at week 4 p = 0.036. Reduction of allodynia p = 0.03. Reduction of cold pain thresholds p = 0.029. Improvements of neuropathic symptoms and quality of life p<0.05.  **Certainty of Evidence:** Moderate. The randomized, double-blind design is a strength. However, the relatively small sample size and the dropout rate reduce the certainty of the evidence. | **Dosage/Injection BTX:** 100U/vial BTX-A reconstituted with 4ml saline solution (5 units BTX-A/0.2ml). Intradermal injections, 0.2ml (5 units BTX-A) per site, 1.5cm apart, maximum 40 sites (200 units).  **Efficacy of BTX:** BTX-A demonstrated significant analgesic effects in patients with focal chronic neuropathic pain, reducing pain intensity and allodynia, and improving quality of life. |
| Wu et al. 2012 [76]  **Location:** The First Affiliated Hospital of Zhengzhou University **Country:** China | To assess the effectiveness and safety of BTX-A in treating patients with TN. | **Study Design:** RCT.  **Size:** 42 patients. BTX-A group: 22 patients. Placebo group: 20 patients. **Age:** Mean age 58.60 (SD 14.62) years. **Sex:** 13 females in the BTX-A group, 10 females in the placebo group.  **Diagnosis:** Classical TN, diagnosed according to the International Classification of Headache Disorders.  **Treatment Period:** 13 weeks (1-week observation, 12-week double-blind period). | Primary: Pain severity (11-point VAS), pain attack frequency per day. Secondary: PGIC. Proportion of responders (≥50% reduction in mean pain score). Safety (adverse events).  Short-term facial asymmetry in 5 patients in the BTX-A group. Transient oedema at the injection site in 3 patients (2 BTX-A, 1 placebo). Two patients discontinued due to lack of efficacy. | BTX-A led to a notable decrease in average VAS scores versus placebo, suggesting successful pain alleviation. In addition, BTX-A resulted in a notable reduction in the occurrence of pain attacks compared to placebo. A greater proportion of patients in the BTX-A group indicated improvement on the PGIC scale, showing a favorable reaction to the treatment. Moreover, a notably larger percentage of patients in the BTX-A group experienced a ≥50% reduction in pain, further validating the treatment's effectiveness. | **Effect Size:** Significant reduction in VAS scores (BTX-A vs. placebo): p < 0.05. Significant reduction in attack frequency (BTX-A vs. placebo): p < 0.05. Significant improvement in PGIC scores (BTX-A vs. placebo): p < 0.01. Significant higher responce rate in the BTX-A group vs the placebo group: P<0.01.  **Certainty of Evidence:** Moderate. The randomized, double-blind, placebo-controlled design is a strength. However, the relatively small sample size may limit the generalizability of the findings. | **Dosage/Injection BTX:** 75 U BTX-A (1.5 mL). 15 injection points, 5 U (0.1 mL) per point. Intradermal and/or submucosal injections.  **Efficacy of BTX:** BTX-A was effective in significantly reducing pain intensity and attack frequency in patients with TN, and was well tolerated. |
| Turk et al. 2005 [77]  **Location:** Dr. Lu¨tfi Kırdar, Kartal Research and Training Hospital **Country:** Turkey | To examine the effectiveness of BTX injections in instances of refractory TN. | **Study Design:** RCT.  **Size:** 8 patients. Intervention group: 8 patients. Control group: None (open-ended study). **Age:** Mean age 57.1 ± 10.1 years. **Sex:** 2 men, 6 women.  **Diagnosis:** Idiopathic TN refractory to medical treatment.  **Treatment Period:** 6 months. | Frequency of pain.  Severity of pain (VAS).  One patient developed dysesthesia for 1 week. One patient reported difficulty in chewing for 3 to 4 days. No serious treatment-related adverse events were reported. | The injection of BTX led to a notable reduction in both the occurrence and intensity of pain, presenting encouraging results for patients. This drug has demonstrated effectiveness in managing trigeminal neuralgia, delivering significant relief. Crucially, no major negative effects were noted in the patients, indicating that the treatment is typically well-accepted and secure. | **Effect Size:** Comparison of VAS stages: P = 0.011 (baseline vs. week 1, month 2, and 6), P = 0.027 (week 1 vs. month 2), P = 0.010 (week 1 vs. month 6), P = 0.034 (month 2 vs. month 6). Comparison of pain frequency: P = 0.012 (baseline vs. week 1, month 2, and 6), P = 0.017 (week 1 vs. month 2), P = 0.011 (week 1 vs. month 6).  **Certainty of Evidence:** Very Low. The study had a very small sample size and was an open-ended study without a control group, significantly limiting the certainty of the evidence. | **Dosage/Injection BTX:** 100 U BTX (BTX-A, Allergan BOTOX) reconstituted in 2 mL saline. 50 U injected at two points on the zygomatic arch.  **Efficacy of BTX:** BTX was found to be effective in reducing pain frequency and severity in patients with refractory TN. |
| Yuan et al. 2009 [78]  **Location:** Taipei Medical University Hospital **Country:** Taiwan | To evaluate the effects of BTX-A injections on pain symptoms in patients with DSPN. | **Study Design:** RCT.  **Size:** 20 patients (18 completed the trial). Intervention group: 10 BTX-A injections. Control group: 8 Normal saline injections (placebo). **Age:** Mean age 65.6 ± 9.2 years. **Sex:** 6 males, 12 females.  **Diagnosis:** Type 2 diabetes with neuropathic pain in both feet, diagnosed using the DN4 questionnaire and nerve conduction velocity examinations.  **Treatment Period:** 6 months (12 weeks per treatment period, with a crossover). | Primary: VAS for pain.  Secondary: CPSQI, SF-36 quality-of-life questionnaire.  One case of mild local skin infection at the injection site. No other significant adverse events reported. | BTX-A led to a marked decrease in VAS pain scores relative to placebo, showcasing its efficacy in relieving pain. Moreover, BTX-A resulted in better sleep quality, according to the CPSQI, at 4 weeks after treatment. Nonetheless, in spite of these beneficial impacts on pain and sleep, BTX-A did not notably enhance quality-of-life scores on the SF-36 scale, suggesting that while there was improvement in pain relief and sleep quality, other wider aspects of quality of life may not have been as significantly influenced. | **Effect Size:** VAS reduction (BTX-A vs. placebo): p = 0.014 (4 weeks), p = 0.039 (8 weeks), p = 0.024 (12 weeks). CPSQI improvement (BTX-A vs. placebo, 4 weeks): p = 0.04. VAS reduction of greater than 3 units, BTX-A vs Placebo: p=0.005  **Certainty of Evidence:** Moderate. The randomized, double-blind, crossover design is a strength. However, the relatively small sample size and specific patient population limit the generalizability. | **Dosage/Injection BTX:** 50 units of BTX-A in 1.2 mL 0.9% saline per foot. Approximately 4 units BTX-A (0.10 mL) per injection site, 12 injection sites per foot. Intradermal injections.  **Efficacy of BTX:** BTX-A was effective in significantly reducing pain intensity in diabetic neuropathic pain, and showed transient improvement in sleep quality. |
| Breuer et al. 2006 [79]  **Location:** New York University School of Medicine **Country:** Unites States | To carry out a pilot study assessing the effectiveness of BTX-B in alleviating hand pain and discomfort linked to CTS. | **Study Design:** RCT.  **Size:** 20 patients. BTX-B group: 11 patients. Placebo group: 9 patients. **Age:** 18-90 years. **Sex:** not specificated.  **Diagnosis:** Mild to moderate CTS confirmed by electromyography-nerve conduction velocity and clinical diagnosis based on a positive Tinel's sign.  **Treatment Period:** 13 weeks. | WHYMPI. NRS for overall pain intensity, pain interference with sleep, and CTS-related tingling. Daily pain diaries.  Two patients experienced weakness, stiffness, and discomfort in the fourth and fifth fingers after receiving higher doses of BTX-B. These adverse events resolved completely within 5-6 weeks. The lower dose of BTX B used for the rest of the study did not produce these side effects. | No statistically significant difference was observed in pain relief between the BTX B group and the placebo group, indicating that BTX-B might not provide a distinct benefit compared to the placebo. Nonetheless, both groups demonstrated symptom enhancement, suggesting a significant potential placebo effect affecting the outcomes. The greatest enhancement for both groups was noted between weeks 6 and 9, emphasizing a period where the most significant alterations took place for both treatments. | **Effect Size:** Overall pain per phone: p = 0.24; Overall pain per diary: p = 0.06; Sleep disturbance per phone: p = 0.62; Sleep disturbance per diary: p = 0.01; CTS-related nighttime tingling: p = 0.40; CTS-related daytime tingling: p = 0.37; Interference with WHYMPI quality of life composite score: p = 0.02  **Certainty of Evidence:** Moderate to Low. Due to the small sample size, and the large placebo effect, the certainty of the evidence is reduced. | **Dosage/Injection BTX:** Initial protocol: 2,500, 5,000, or 7,500 units of BTX B. Revised protocol: 2,500 units of BTX-B. Injected into the Opponens Digiti Minimi, Flexor Digiti Minimi, and Palmaris Brevis muscles. Injection volume: 0.5 ml, equally distributed into the 3 muscles.  **Efficacy of BTX:** BTX B was not found to be significantly more effective than placebo in relieving pain and discomfort associated with CTS. |
| Safarpour et al. 2010 [80]  **Location:** Yale **Country:** United States | To examine the effectiveness and tolerability of BTX-A in treating allodynia in patients with CRPS. | **Study Design:** RCT.  **Size:** 14 patients. Double-blind study: 4 BTX-A, 4 placebo. Open-label study: 6 BTX-A. **Age:** Mean age 47.12 years (range 37–55) in the double blind study. **Sex:** 5 females out of the 8 patients in the double blind study. Sex distribution was not given for the open label portion of the study.  **Diagnosis:** CRPS meeting the International Association for the Study of Pain diagnostic criteria.  **Treatment Period:** Baseline, 3 weeks, and 2 months after BTX-A administration. | Brief pain inventory. McGill Pain Questionnaire.  Clinical Pain Impact Questionnaire. Quantitative skin sensory test. Sleep satisfaction scale.  Patient global satisfaction scale.  The treatment was painful and poorly tolerated. | None of the allodynia patients exhibited a notable reaction to BTX-A therapy, suggesting that this specific condition might not gain much from the treatment. Furthermore, the treatment was painful and not well-accepted by the patients, indicating it may not be appropriate for everyone, especially for those with increased sensitivity or discomfort. | **Effect Size:** P values for pain intensity and pain days were not significant (P > 0.05). P values for the secondary outcomes also failed to show significant improvement.  **Certainty of Evidence:** Very Low. The study had a very small sample size, and the open label portion of the study is particularly subject to bias. The double blind portion of the study was also very small. | **Dosage/Injection BTX:** 5 units/site, intradermally and subcutaneously. Total dose 40–200 units (mean: 79.5).  **Efficacy of BTX:** BTX-A administered intradermally and subcutaneously failed to improve pain and was poorly tolerated in patients with CRPS-associated allodynia. |
| Carroll et al. 2009 [81]  **Location:** Stanford University School of Medicine, Palo Alto Veterans Affairs Hospital **Country:** United States | To evaluate the length of analgesia following a standard LSB using bupivacaine versus LSB with bupivacaine and BTX-A in individuals with CRPS. | **Study Design:** RCT.  **Size:** 9 patients. **Age:** not specificated. **Sex:** Predominantly female.  **Diagnosis:** CRPS type I, meeting the International Association for the Study of Pain criteria.  **Treatment Period:** Patients received two LSB injections, with a one-month interval between injections. | Primary: Time to return to baseline pain (analgesic failure).  Secondary: Change in VAS score over time.  One patient experienced significant nausea and emesis, which resolved spontaneously. | BTX-A significantly extended the pain relief after sympathetic block, increasing the length of analgesia. The median duration until analgesic failure was notably extended following LSB combined with BTX-A compared to LSB with bupivacaine only, demonstrating the additional advantage of BTX-A in maintaining pain relief. Additionally, the BTX-A -augmented sympathetic blockade resulted in a notable decrease in VAS pain scores over time, showcasing its efficacy in delivering prolonged pain relief. | **Effect Size:** Log-rank test for analgesic failure: p < 0.02. Mixed-effects model for VAS reduction: p < 0.0001.  **Certainty of Evidence:** Moderate. The randomized, double-blind, crossover design is a strength. However, the small sample size and the predominantly female population limit the generalizability of the findings. | **Dosage/Injection BTX:** 75 units of BTX-A mixed with 10 ml 0.5% bupivacaine. Lumbar sympathetic block injection.  **Efficacy of BTX:** BTX-A significantly prolonged the analgesic effects of LSB in patients with CRPS. |
| Wu et al. 2012 [82]  **Location:** Medical College of Wisconsin, Milwaukee, WI  **Country:** United States | To examine the efficacy of BTX-A (Botox) and Lidocaine/Depomedrol injections in managing RLP and PLP among amputees. | **Study Design:** RCT.  **Size:** 14 patients. Botox group: 7 patients. Lidocaine/Depomedrol group: 7 patients. **Age:** Adult amputees (18 or more years old). **Sex:** not specificated.  **Diagnosis:** Lower extremity amputees with a clinical diagnosis of daily RLP and/or PLP greater than 5/10 on the VAS score.  **Treatment Period:** 6 months. | Changes in intensity of RLP and PLP as recorded by VAS.  Changes in pressure pain tolerance as determined by a pressure algometer.  No specific adverse events. | Both Botox and Lidocaine/Depomedrol injections led to prompt enhancements in RLP and pain tolerance, indicating that both therapies can offer quick relief for these symptoms. Nonetheless, no notable enhancements in PLP were seen in either group, suggesting that neither treatment was especially effective for this kind of pain. Significantly, Botox indicated a tendency for enhanced improvement in RLP when compared to Lidocaine/Depomedrol, especially in the 3-6 month timeframe, implying that Botox could provide more enduring relief as time passes. | **Effect Size:** RLP improvement (Botox): P = 0.002. RLP improvement (Lidocaine/Depomedrol): P = 0.06. Pain tolerance improvement (Botox): P = 0.01. Pain tolerance improvement (Lidocaine/Depomedrol): P = 0.07. No significant improvement of PLP in either group.  **Certainty of Evidence:** Low. The pilot study design and small sample size limit the certainty of the evidence. However, the prospective, double-blind, randomized design provides some strength. | **Dosage/Injection BTX:** 50 units of Botox per injection site, total units ranging from 250 to 300 units. Injected intramuscularly and cutaneous/subcutaneously.  **Efficacy of BTX:** Botox injections showed significant improvement in RLP and pain tolerance, but not in PLP, in amputees. It also showed a trend towards greater RLP improvement than Lidocaine/Depomedrol. |
| Zuniga et al. 2013 [83]  **Location:** Hospital de Clínicas, University of Buenos Aires **Country:** Argentina | To evaluate the effectiveness and safety of a one-time injection of BTX-A (Botox) for treating pain in ETN. | **Study Design:** RCT.  **Size:** 36 patients. Botox (BTX) group: 20 patients. Placebo (0.9% saline) group: 16 patients. **Age:** Older than 18 years. **Sex:** 19 men and 17 women.  **Diagnosis:** ETN based on established clinical criteria, confirmed by brain magnetic resonance imaging to rule out secondary causes.  **Treatment Period:** 3 months (assessments at baseline, 1, 2, and 3 months post-injection). | VAS for pain intensity. Impact on function in activities of daily living. SF36 for quality of life. Frequency of attacks per day.  Hematoma at the injection site (2 BTX patients). Slight facial asymmetry due to weakness (2 BTX patients). | BTX substantially lowered pain intensity (VAS) and attack frequency compared to placebo, with the greatest enhancements seen at 2 and 3 months following treatment. When BTX was included in current medication plans, a combined pain reduction effect was noticeable, indicating that the combination of therapies might provide improved advantages. Nonetheless, no substantial differences were found in quality of life (SF36) among the groups, suggesting that although BTX successfully alleviated pain, it did not lead to notable enhancements in the wider dimensions of well-being evaluated by the SF36. | **Effect Size:** Significant reduction in paroxysms at 1, 2, and 3 months (p = 0.036, p = 0.01, p = 0.006, respectively). Significant reduction in VAS pain scores at 3 months (p = 0.01). Cox proportional risk model for relapse in BTX group: p=0.017.  **Certainty of Evidence:** Moderate. The double-blind, randomized, placebo-controlled design is a strength. However, the sample size, while reasonable, could be larger, and longer term follow up would have been beneficial. | **Dosage/Injection BTX:** 50 U of BTX subcutaneously. 10 U of BTX intramuscularly in the masseter muscle (for patients with V3 involvement). Multiple subcutaneous injections, 1 cm apart, along the path of the affected trigeminal nerve branches.  **Efficacy of BTX:** BTX demonstrated significant efficacy in reducing pain severity and attack frequency in ETN patients, especially when added to their existing medication regimens. |
| Shebata et al. 2013 [84]  **Location:** Cairo University **Country:** Egypt | To evaluate the efficacy and safety of BTX-A in the treatment of intractable idiopathic TN. | **Study Design:** RCT.  **Size:** 20 patients. BTX-A group: 10 patients. Placebo group: 10 patients. **Age:** 27 to 72 years (mean age: 45.95 ± 10.02 years). **Sex:** 9 males (45%) and 11 females (55%).  **Diagnosis:** Idiopathic TN according to IHS criteria, intractable to medical treatment.  **Treatment Period:** 12 weeks. | Pain severity reduction using VAS.  Paroxysms frequency. Quality of life scale.  Number of weekly acute medications.  Facial asymmetry. Hematoma at the injection site. Itching at the injection site. Pain at the injection site. All adverse events were transitory and mild. | BTX-A considerably diminished pain severity (VAS) and the rate of paroxysms in comparison to placebo, showing its efficacy in reducing both the intensity and frequency of pain. Moreover, BTX-A was shown to notably enhance quality of life, suggesting additional advantages beyond just alleviating pain. Moreover, BTX-A notably reduced the weekly intake of acute medications, indicating that it not only alleviates pain but also diminishes the necessity for further pharmacological treatments. | **Effect Size:** Pain reduction (VAS): P < 0.0001. Paroxysms frequency reduction: P < 0.0001. Quality of life improvement: P < 0.0001. Reduction of acute medication use: P<0.0001  **Certainty of Evidence:** Low. The randomized, double-blind, placebo-controlled design is a strength. However, the relatively small sample size slightly reduces the certainty of the evidence. | **Dosage/Injection BTX:** 100 U Botox in 2 mL preservative-free normal saline (5 units/0.1 mL). 5 units per injection point, subcutaneously. Total dosage ranged from 40 to 60 units.  **Efficacy of BTX:** BTX-A was found to be effective in reducing pain severity, paroxysms frequency, and improving quality of life in patients with intractable TN. |
| Spruijt et al. 2024 [85]  **Location:** four Dutch hospital clinics **Country:** Netherlands | To assess the effectiveness of BTX-A injections versus placebo in women suffering from CPP. | **Study Design:** RCT.  **Size:** 94 patients. BTX-A group: 47 women. Placebo group: 47 women. **Age:** Median age 47 years (intervention group), 45 years (placebo group). **Sex:** All participants were female.  **Diagnosis:** CPP according to ICS criteria, combined with increased muscle tension despite PFMT.  **Treatment Period:** 26 weeks. | Primary: 33% reduction in pelvic pain (painDETECT) and PGI-I. Secondary: VAS pain scores, PFDI-20, PFIQ-7, EQ-5D, PCS, HADS, PISQ-IR, and pelvic floor muscle activity (MAPLe).  Immediate post-treatment bleeding (4% in each group). Mild bleeding in the first week (8% BTX-A, 2% placebo; p = 0.053). Stress urinary incontinence (1 participant, BTX-A group). Constipation (3 participants, BTX-A group) Severe distress and intense pain (1 participant, BTX-A group) | There was no notable difference in effectiveness between BTX-A and placebo injections for alleviating CPP. Each group showed comparable enhancements in average pain scores throughout the 26-week span. Nonetheless, a notable reduction in pelvic floor resting activity was identified in the BTX-A group, as assessed by the MAPLe. | **Effect Size:** Primary outcome (average pain score reduction): p = 0.19. Primary outcome (PGI-I): p = 0.92. MAPLe outcomes (Pelvic floor resting activity): p <= 0.001.  **Certainty of Evidence:** Moderate. The randomized, double-blind, placebo-controlled design is a strength. However, the possible placebo effect and other factors influencing the placebo group's improvement reduce the certainty of evidence. | **Dosage/Injection BTX:** 100 units of BTX-A dissolved in 6 mL saline. Injected into the m. puborectalis and/or m. pubococcygeus (2–6 sites, 1–3 mL per site).  **Efficacy of BTX:** BTX-A injections were not significantly more effective than placebo injections in the management of CPP in women. |
| Restivo et al. 2018 [31]  **Location:** Catania, Garibaldi-Nesima Hospital **Country:** Italy | To assess the effectiveness and safety of BTX-A for managing muscle cramps in individuals with DN. | **Study Design:** RCT.  **Size:** 50 patients. BTX-A group: 25 patients. Placebo group: 25 patients. **Age:** Mean age 63.4 ± 8.5 years. **Sex:** BTX-A group: 12 females and 13 males. Placebo group: 11 females and 14 males  **Diagnosis:** Type 2 diabetes with DN, confirmed by clinical and electrophysiological criteria, and experiencing calf/foot cramps.  **Treatment Period:** 20 weeks. | Primary: Cramping pain intensity (Brief Pain Inventory-Modified Short Form). Secondary: Number of cramp episodes, CSS, CTF.  BTX-A was well tolerated. Mild pain at the injection site was reported in 4 BTX-A patients and 3 placebo patients, resolving within 3 days. No systemic adverse events or motor weakness was reported. | BTX-A notably decreased the intensity of cramp pain, the frequency of cramp episodes, and the CSS, in addition to raising the CTF when compared to a placebo. The impact of BTX-A became noticeable as soon as one week after the injection and persisted for 14-16 weeks. | **Effect Size:** Significant improvements in all outcome measures in the BTX-A group compared to baseline and placebo (p < 0.05). Significant differences were seen between the groups from week 2 to week 14 for pain intensity.  **Certainty of Evidence:** Moderate. The randomized, double-blind, placebo-controlled design is a strong factor. However, the single-center study and moderate sample size slightly reduce the certainty. | **Dosage/Injection BTX:** 100 units of incobotulinumtoxinA (Xeomin) in 1ml saline for gastrocnemius muscle injections (0.5ml per side). 30 units of incobotulinumtoxinA in 1ml saline for foot flexor muscle injections (0.15ml per side). Placebo group received saline injections.  **Efficacy of BTX:** BTX-A was effective in significantly reducing muscle cramps and associated pain in patients with DN, with a clinically relevant and sustained effect. |
| Al-Awady et al. 2025 [86]  **Location:** Icahn School of Medicine at Mount Sinai, a large urban medical center **Country:** United States | To assess the therapeutic effectiveness of BoNTA at various time points after the onset of symptoms in individuals with synkinesis resulting from Bell's Palsy. | **Study Design:** Retrospective Study  **Size:** 63 patients. Group 1 (6-12 months): 28 patients. Group 2 (13-24 months): 14 patients. Group 3 (25+ months): 23 patients. **Age:** Average age 54 years. **Sex:** 81% female overall.  **Diagnosis:** Synkinesis as a sequelae of Bell's Palsy.  **Treatment Period:** Variable, patients were grouped based on the timing of BoNTA treatment post-symptom onset: 6-12 months, 13-24 months, and after 24 months. | HB, FaCE, SAQ, FGS.  No specific adverse events. | FGS scores showed a significant enhancement when BoNTA was given 6 to 12 months after symptoms began. Nonetheless, there were no noteworthy differences noted among groups in HB, FaCE, and SAQ scores. The analysis indicated a statistically significant difference in the number of BoNTA treatments and the time elapsed between the last treatment and follow-up across groups. | **Effect Size:** FGS score change: P = 0.03 (significant difference between groups). HB score change: P = 0.91 (no significant difference). FaCE score change: P = 0.11 (no significant difference). SAQ score change: P = 0.28 (no significant difference). No statistically significant differences were observed between the baseline scores for the FGS(P = .94).  **Certainty of Evidence:** Low. The retrospective design limits the certainty of evidence. | **Dosage/Injection BTX:** not specificated.  **Efficacy of BTX:** BoNTA was shown to be effective in improving FGS scores, especially when administered between 6 and 12 months post-symptom onset. |
| Taylor et al. 2008 [87]  **Location:** Ohio **Country:** United States | To assess the effectiveness of BTX-A occipital nerve blocks in alleviating ON-related pain and paresthesias, and to examine its influence on headache-free days, medication consumption, and overall quality of life. | **Study Design:** prospective open-label study  **Size:** 6 subjects **Age:** 24 and 53 years. **Sex:** 2 men and 4 women.  **Diagnosis:** Chronic ON according to the International Headache Society criteria.  **Treatment Period:** 12 weeks post-treatment, with a 2-week baseline period. | Primary: VPAM for pain intensity. Secondary: Headache-free days, medication use (VPAM), MOS-SF36, BDI, HDI, HSQL.  No specific adverse events. | The study reported significant improvements in sharp and shooting pain, while dull aching pain and pins-and-needles symptoms showed non-significant trends toward improvement. HSQL scores improved significantly. No statistically significant changes were observed in BDI, HDI, or the mental health domain of the MOS-SF36, although each showed slight positive trends. In contrast, the physical health domain of the MOS-SF36 demonstrated a significant improvement by the end of the study. Despite these gains, there was no reduction in daily rescue medication use, and participants did not report any headache-free days. | **Effect Size:** Sharp/shooting pain: Statistically significant improvement (P < 0.05). HSQL: Statistically significant improvement (P = 0.0211 and P = 0.0315). MOS-SF36 Physical Health: Statistically significant improvement (P = 0.0300).  **Certainty of Evidence:** Very Low. The study had a very small sample size and lacked a control group (placebo), which significantly limits the certainty of evidence. | **Dosage/Injection BTX:** 50 units of BTX-A reconstituted in 3 cc of normal saline injected at each symptomatic side.  **Efficacy of BTX:** BTX-A occipital nerve blocks showed some efficacy in reducing sharp/shooting pain and improving headache-specific quality of life in patients with ON. |
| Tsai et al. 2006 [88]  **Location:** Taipei Veterans General Hospital **Country:** Taiwan | To evaluate the effectiveness and safety of BTX-A in enhancing clinical symptoms, signs, and motor function in CTS. | **Study Design:** Uncontrolled Experimental Study  **Size:** 5 patients. **Age:** Mean age 52.2 ± 2.5 years. **Sex:** 5 female patients.  **Diagnosis:** CTS.  **Treatment Period:** 3 months. | VAS for pain. Electrophysiological studies for conduction time between wrist and palm.  No adverse reactions were observed. | Electrophysiological investigations showed no statistically meaningful differences prior to and following treatment. Nonetheless, regarding pain perception, three patients indicated an improvement in their VAS pain scores, while one reported a decline and another exhibited no change. While the VAS data suggested a trend for improvement (p = 0.2), it fell short of achieving statistical significance. Significantly, no negative reactions were noted during the study. | **Effect Size:** Electrophysiological studies: No significant differences. VAS scores: p = 0.2 (trend toward improvement).  **Certainty of Evidence:** Low. The study had a very small sample size and lacked a control group (placebo), which significantly limits the certainty of evidence. | **Dosage/Injection BTX:** 30 units of BTX-A on each side of the carpal tunnel.  **Efficacy of BTX:** BTX-A demonstrated a tendency for alleviating pain symptoms in patients with CTS, although there was no enhancement in the electrophysiological function. The findings are not definitive because of the limited sample size and absence of a control group. |
| Gaber et al. 2022 [89]  **Location:** not specificated. **Country:** Egypt. | To evaluate the effectiveness of supplemental duloxetine, gabapentin, and intradermal BTX-A injections in alleviating pain and enhancing sleep quality in individuals with DPN who are already receiving carbamazepine. | **Study Design:** Comparative Study.  **Size:** Duloxetine group (add-on): 10 patients. Gabapentin group (add-on): 10 patients. BTX-A group (intradermal injection): 10 patients. **Age:** Mean age approximately 59-63 years across the three groups. **Sex:** Duloxetine (n=10): 5 males (50%), 5 females (50%); Gabapentin (n=10): 4 males (40%), 6 females (60%); Botox (n=10): 4 males (40%), 6 females (60%).  **Diagnosis:** Type 2 diabetes mellitus with DPN, confirmed by nerve conduction study.  **Treatment Period:** 12 weeks. | VAS and PSQI.  No specific adverse events. | The three treatments—duloxetine, gabapentin, and BTX-A resulted in noteworthy decreases in VAS scores and enhancements in PSQI scores. Nonetheless, BTX-A exhibited the quickest and most significant effects, attaining the highest decreases in both metrics. Importantly, it was the sole intervention that consistently yielded statistically significant enhancements in VAS and PSQI scores over the full 12-week research duration. | **Effect Size:** Significant reduction in VAS and PSQI scores for all three groups (p < 0.001). BTX-A showed statistically significant differences versus the other two groups in PSQI scores at the 4 week follow up (p=0.002 comparing to duloxetine, and p=0.005 comparing to gabapentin). BTX-A caused statistically significant differences in VAS and PSQI scores all along the study duration (p<0.05).  **Certainty of Evidence:** Low. The randomized design strengthens the evidence, but the relatively small sample size and single-center study limit the certainty. | **Dosage/Injection BTX:** 50 units of BTX-A intradermal, evenly distributed in 10 injection sites per foot.  **Efficacy of BTX:** BTX-A proved effective in alleviating pain and enhancing sleep quality in individuals with DPN, demonstrating greater efficacy than duloxetine and gabapentin in this research. |
| Tereshko et al. 2023 [90]  **Location:** not specificated. **Country:** Italy | To assess how TN patients react to BTX-A and to analyze the treatment response of type 1 and type 2 TN patients to BTX-A. | **Study Design:** Prospective Observational Study.  **Size:** Intervention group: 40 patients (no control group). Type 1 TN: 18 patients. Type 2 TN: 22 patients. **Age:** Mean age 63.4 ± 18.4 years. **Sex:**  **Diagnosis:** TN, subclassified into type 1 (paroxysmal pain) and type 2 (continuous pain with paroxysms).  **Treatment Period:** 3 months, follow-up. | VAS for pain intensity. Paroxysm frequency (number of episodes per week). PFPS for quality of life. PGIC.  Facial asymmetry (mild and transient) was reported in 14 patients. | BTX-A treatment resulted in a notable enhancement in VAS scores and a decrease in paroxysm frequency among all patients with TN. Patients with both type 1 and type 2 TN experienced similar advantages, demonstrating alike decreases in pain intensity, frequency of paroxysms, and PFPS scores. No major differences were noted in treatment response among the two TN subtypes. The main negative outcome noted was facial asymmetry. | **Effect Size:** Significant improvement in VAS scores (p < 0.001). Significant reduction in paroxysm frequency (p < 0.001). Significant improvement in PFPS scores (p < 0.001). No significant difference between type 1 and type 2 TN in pain and paroxysm frequency reduction. Significant correlation between baseline paroxysm pain and background pain in type 2 TN (p<0.001).  **Certainty of Evidence:** Moderate. The prospective design strengthens the evidence, but the lack of a control group and the relatively small sample size limit the certainty. | **Dosage/Injection BTX:** Mean BTX-A doses were 29.7 ± 11.4 U for type 1 TN and 29.3 ± 11.1 U for type 2 TN.  **Efficacy of BTX:** BTX-A successfully reduced pain and the frequency of paroxysms in patients with type 1 and type 2 TN, showing comparable efficacy in both types. |
| Laterza et al. 2024 [91]  **Location:** not specificated. **Country:** Italy | To assess the impact of ongoing BTX-A treatment for neuropathic pain over a duration of 1 year. | **Study Design:** Prospective Study.  **Size:** 32 patients **Age:** Mean age 58 ± 27 years. **Sex:** 19 females and 13 males.  **Diagnosis:** TN (31%); PHN (9%); Mixed peripheral neuropathic pain (41%); Central neuropathic pain (19%).  **Treatment Period:** 1 years. | 11-point NRS of the BPI. NPSI.  No serious side effects were observed. | Frequent subcutaneous injections of BoNT-A led to a marked decrease in pain intensity in all NP groups. Significantly, no major side effects were noted. | **Effect Size:** Significant reduction in NRS scores for each group (P < 0.01). Central neuropathic pain group t(5) = [3.37], P < 0.01; mixed peripheral neuropathic pain group t(11) = [6.5], P < 0.01; post-herpetic neuralgia t(2) = [5.5], P < 0.01; TN group t(9) = [6.7], P < 0.01.  **Certainty of Evidence:** Low. The prospective design strengthens the evidence, but the lack of a control group and the relatively small sample size limit the certainty. | **Dosage/Injection BTX:** Up to 300 units (U) of BTX-A, administered subcutaneously in several injections, spaced 4 months apart (3 total treatments during the one-year period).  **Efficacy of BTX:** Repeated subcutaneous BTX-A injections were safe and effective in reducing pain intensity in patients with chronic neuropathic pain. |
| Bohluli et al. 2011 [92]  **Location:** not specificated. **Country:** Tehran | To evaluate the effectiveness of BTX-A on TN. | **Study Design:** Open-label, prospective study.  **Size:** 15 patients. **Age:** Between 28 and 67 years (mean 48.9 years). **Sex:** 8 men and 7 women.  **Diagnosis:** Clinically documented TN according to Winn criteria and International Headache Society criteria.  **Treatment Period:** 6 months follow-up. | Frequency of TN attacks (number per day). Severity of pain (11-point visual analog scale - VAS). Patient global assessment scale (9-point).  Transient paresis of the buccal branch of the facial nerve in 3 patients. | Every patient showed enhancements in the occurrence and intensity of pain episodes. Significantly, seven patients experienced total pain relief, whereas five only needed nonsteroidal anti-inflammatory medications for pain management. Moreover, three patients who were previously unresponsive to therapy became responsive to anticonvulsant medications. The sole noted adverse effect was temporary weakness of the buccal branch of the facial nerve, observed in three patients. | **Effect Size:** Significant difference in frequency of attacks before and after injection (P < 0.001). Significant difference in severity of pain before and after injection (P < 0.001). Significant improvement in patient global assessment scale (P < 0.001).  **Certainty of Evidence:** Low. The open-label design and small sample size limit the certainty. | **Dosage/Injection BTX:** 50 U of reconstituted BTX-A solution injected at the trigger zones.  **Efficacy of BTX:** BTX-A was found to be effective in reducing the frequency and severity of pain attacks in patients with TN. |
| Piovesan et al. 2005 [93]  **Location:** not specificated. **Country:** Brazil. | To document the application of BTX-A for treating TN, identifying the least dose necessary and the length of its effect. | **Study Design:** Open-label, prospective study.  **Size:** 13 patients. **Age:** Men: average 67.75 ± 6.6 years; Women: average 59.22 ± 14.26 years. **Sex:** 4 men and 9 women.  **Diagnosis:** TN according to the IHS criteria.  **Treatment Period:** 60 days follow-up. | Analgesic time effect of BTX-A. Intensity of pain (visual analog scale - VAS). Number and distribution of paroxysmal pain. Consumption of analgesics.  Three patients had asymmetrical facial wrinkles. One patient had slight eyelid ptosis. | BTX-A injections resulted in a notable decrease in the area and intensity of pain. Furthermore, there was a significant reduction in the demand for preventive medication. The therapeutic benefits of BTX-A lasted for over 60 days. | **Effect Size:** Significant reduction of the pain for 10 days, patients were almost symptom free at 20 days, and there was a slight increase at 60 days. (p < 0.05)  **Certainty of Evidence:** Low. The open-label design and small sample size limit the certainty. | **Dosage/Injection BTX:** The dose of BTX-A varied for each patient, depending on the reported pain surface. Injections were performed transcutaneously (subdermal). The mean TC for all branches and all patients was 3.22 units/cm2.  **Efficacy of BTX:** BTX-A was shown to be successful in diminishing both the area and severity of pain in individuals with TN. |
| Borodic et al. 2002 [94]  **Location:** Massachusetts  **Country:** United States | To assess the effectiveness of BTX injections in treating patients with chronic facial pain who are seeking specialized care at a pain clinic. | **Study Design:** Open-label pilot study.  **Size:** 44 patients. **Age:** Average 54.2 years (range, 34 to 89 years). **Sex:** 12 men and 32 women.  **Diagnosis:** Chronic facial pain, including: Temporomandibular joint syndrome; postsurgical pain syndromes; Essential headache; Idiopathic TN.  **Treatment Period:** Average follow-up of 7.65 months (range, 4 to 20 months). | Patient perception of at least 50% reduction in pain frequency and/or intensity. Reduction in analgesic medication use. Patient desire for further injections. Categorical assignment of responder or nonresponder status.  Temporary facial asymmetry. Weakness secondary to neuromuscular effects of BTX. | Of the 44 patients who received BTX injections, 33 (75%) showed a positive response. Specifically, 8 of the 11 patients suffering from TN exhibited a favorable response. The length of the positive impacts differed, extending from 2 to 4 months. The reported complications were minor, mainly involving temporary facial asymmetry and weakness. Notably, facial swelling and redness seemed to indicate treatment response effectively. | **Effect Size:** Inflammatory signs showed a statistically significant correlation with treatment response(P < .01). No significant difference in response rates among the diagnosis groups or between neuropathic and myofascial pain types.  **Certainty of Evidence:** Low. The open-label design and lack of a control group limit the certainty. | **Dosage/Injection BTX:** Doses ranged from 25 to 75 LD50 units of Hall strain–derived BTX-A. Maximum of 7.5 units per percutaneous puncture. Injection sites were tailored to the location of the pain.  **Efficacy of BTX:** BTX injections proved effective in alleviating chronic facial pain for a considerable number of the patients examined. |
| Alizadeh et al. 2023 [95]  **Location:** not specificated **Country:** Iran | To investigate the effect of BTX-A injection on phantom pain in patients. | **Study Design:** Double Blind Clinical Trial.  **Size:** 30 patients. Intervention group (A): 15 patients. Placebo group (B): 15 patients. **Age:** Group A: Mean age 56.13 years. Group B: Mean age 53.86 years. **Sex:** Group A (intervention): 5 female, 10 male. Group B (placebo): 4 female, 11 male.  **Diagnosis:** phantom pain.  **Treatment Period:** 16 weeks follow-up. | VAS for pain. NPS.  Side effects such as headache, cold symptoms, and injection pain were evaluated, but the specific results were not detailed. | BTX-A significantly decreased phantom pain in comparison to a placebo. Remarkably, the intervention group demonstrated considerable pain decrease at 2, 4, and 8 weeks after treatment. The therapy was shown to be effective in relieving pain associated with different reasons for amputation, such as those caused by war, accidents, and unidentified sources. Nevertheless, certain pain attributes, including hot, cold, and itching pain, were not significantly influenced by the BTX therapy. | **Effect Size:** significant reductions in VAS pain scores in the BTX-A group compared with controls at 2, 4, and 8 weeks (p<0.001, p=0.004, p<0.001). Additional subgroup analyses, stratified by amputation etiology, also demonstrated significant differences at several follow-up points.  **Certainty of Evidence:** moderate | **Dosage/Injection BTX:** 100 units of Xeomin® (incobotulinumtoxinA) dissolved in 1 ml of normal saline. Administered intradermally at the amputation site in 10 points, with 5 syringe lines at each point.  **Efficacy of BTX:** BTX-A was found to be effective in reducing phantom pain in patients. |

*Legend: Botulinum Toxic (BTX), botulinum toxin type A (BTX-A), trigeminal neuralgia (TN), Randomized Controlled Trial (RCT), International Classification of Headache Disorders (ICDH), Visual Analog Scale (VAS), Patient Global Impression of Change (PGIC), Diabetic neuropathy (DN), Douleur Neuropathique 4 (DN4), Neuropathy Pain Scale (NPS), diabetic peripheral neuropathy (DSPN), Neuropathic Pain Scale (NPS), 36-item Short Form Health Survey (SF-36), Pittsburgh Sleep Quality Index (PSQI), Numerical Rating Scale (NRS), Brief Pain Inventory (BPI), Neuropathic Pain Symptom Inventory (NPSI), Hospital Anxiety and Depression Scale (HADS), EuroQol 5-Dimension (EQ-5D), Intraepidermal Nerve Fiber Density (IENFD), Substance P (SP), Calcitonin Gene-Related Peptide (CGRP), bladder instillation therapy (BIT), interstitial cystitis/bladder pain syndrome (IC/BPS), O'Leary-Sant (OLS), Female Sexual Function Index (FSFI), Female Sexual Dysfunction Scale-Revised (FSDS-R), Short-Form 12 (SF-12), Urinary tract infections (UTIs), postherpetic neuralgia (PHN), Standard Deviation (SD), intradermal botulinum toxin type A (BoNT/A), Chinese version of the Pittsburgh Sleep Quality Index (CPSQI), carpal tunnel syndrome (CTS), West Haven-Yale Multidimensional Pain Inventory (WHYMPI), Botulinum toxin type B (BTX-B), complex regional pain syndrome (CRPS), lumbar sympathetic block (LSB), residual limb pain (RLP), phantom limb pain (PLP), essential trigeminal neuralgia (ETN), Headache Society (IHS), chronic pelvic pain (CPP), International Continence Society (ICS), pelvic floor muscle therapy (PFMT), Aberdeen Standard Pain scores (AS pain scores), Pelvic Floor Distress Inventory-20 (PFDI-20), Pelvic Floor Impact Questionnaire-7 (PFIQ-7), EuroQol-5 Dimensions (EQ-5D), Physical Component Summary (PCS), Pelvic Organ Prolapse Incontinence Sexual Questionnaire-IR (PISQ-IR), Muscle Activity Profile of the Pelvic Floor (MAPLe), Patient Global Impression of Improvement (PGI-I), cramp severity score (CSS), cramp threshold frequency (CTF), Botulinum Neurotoxin A (BoNTA), House-Brackmann (HB), Facial Clinimetric Evaluation (FaCE), Synkinesis Assessment Questionnaire (SAQ), Facial Grading System (FGS), occipital neuralgia (ON), Visual Analog Pain and Medication Use Diary (VPAM), Beck Depression Index (BDI), Headache Disability Index (HDI), Headache Specific Quality of Life (HSQL), Medical Outcomes Study 36-Item Short Form Health Survey (MOS-SF36), Penn Facial Pain Scale (PFPS).*
